# From Unsupervised to Zero-Shot: 3D Domain Adaptation for Cryo-Electron Tomography Segmentation

**DOI:** 10.1007/s11263-026-02802-6

**Published:** 2026-04-07

**Authors:** Haoran Li, Xingjian Li, Huan Wang, Jiahua Shi, Huaming Chen, Bo Du, Johan Barthelemy, Daisuke Kihara, Jun Shen, Min Xu

**Affiliations:** 1 School of Computing and Information Technology, University of Wollongong, Wollongong, Australia; 2 ARC Training Centre for Innovative Composites for the Future of Sustainable Mining, University of Wollongong, Wollongong, Australia; 3 Ray and Stephanie Lane Computational Biology Department, Carnegie Mellon University, Pittsburgh, USA; 4 Centre for Nutrition and Food Sciences, The University of Queensland, Brisbane, Australia; 5 School of Electrical and Computer Engineering, University of Sydney, Sydney, Australia; 6 Department of Management, Griffith University, Queensland, Australia; 7 NVIDIA, California, USA; 8 Department of Biological Sciences, Purdue University, Lafayette, USA

**Keywords:** 3D image segmentation, Unsupervised domain adaptation, Zero-shot domain adaptation, Cryo-Electron Tomography, Graph Learning

## Abstract

With the refinement of 3D imaging modality, cryo-electron tomography (cryo-ET) has emerged as a powerful technique for the structural analysis of macromolecular complexes at near-atomic resolution. Recent advancements in volumetric segmentation methods applied to cryo-ET datasets have garnered significant attention within the biomedical sector. However, existing methods rely heavily on manually labeled data, which demands highly specialized expertise, making fully supervised approaches less feasible for cryo-ET images. To address this, a number of unsupervised domain adaptation (UDA) techniques have been developed to improve segmentation network performance using unlabeled data. Nevertheless, directly applying these methods to cryo-ET image segmentation presents two major challenges: 1) the source dataset, usually obtained through simulation, contains a fixed level of noise, while the target dataset, being directly collected from raw-data from the real-world scenario, has unpredictable noise levels; 2) the source data used for training typically consists of known macromolecules, in contrast, the target domain data are often unknown, causing the model to be biased towards those known macromolecules, leading to a domain shift problem. To address such challenges, in this paper, we introduce a voxel-wise unsupervised domain adaptation approach, termed Vox-UDA, specifically for cryo-ET subtomogram segmentation. Vox-UDA incorporates a noise generation module to simulate target-like noises in the source dataset for cross-noise level adaptation, and a denoised pseudo-labeling strategy based on the improved bilateral filter to alleviate the domain shift problem. Additionally, we further consider a scenario that is more in line with the real world, where the target (experimental) dataset might not be accessible during training or has a very small sample size, and we present a voxel-wise zero-shot domain adaptation (ZSDA) approach, named Vox-ZSDA. In Vox-ZSDA, we introduce a self-supervised graph learning strategy to eliminate any dependency on the target data, accompanied by a dynamic graph contrastive learning technique to enhance the model’s sensitivity to macromolecular structures to boost the segmentation performance. More importantly, we construct the first UDA and ZSDA cryo-ET subtomogram segmentation benchmark on three experimental datasets. Extensive experimental results on multiple benchmarks and newly curated real-world datasets demonstrate the superiority of our proposed approach compared to state-of-the-art UDA and ZSDA methods.

## 1 Introduction

Cryo-Electron Tomography (cryo-ET) is a state-of-the-art imaging technique that provides three-dimensional visualizations of biomedical samples in their native, frozen-hydrated state (Oikonomou & Jensen, 2017; Pan et al., 2025). This automated electron tomography technique enables biologists to interpret complex macromolecular structures at high resolution (Wan & Briggs, 2016), playing a crucial role in drug discovery and disease treatment, *e.g.,* the research of SARS-CoV-2 structure and replication (Klein et al., 2020). Recent advances on deep learning research significantly enhance this analysis by automating and improving the accuracy of structural recognition, thereby accelerating scientific discovery and the proper development of medical interventions. Especially for the subtomogram segmentation task (Zhou et al., 2021; Zhu et al., 2021), which defines a 3D segmentation task that aims to mine the meaningful information of the target macromolecular on the voxel-level. However, existing methods heavily rely on manual, which are highly subjective and resource-intensive, so that the effectiveness of those methods had always been questionable.

To tackle the challenges for data annotation, the classical unsupervised domain adaptation (UDA) methods involve transferring the knowledge from labeled source domains to unlabeled target domains (Fan et al., 2024; Liu et al., 2020; Zhao et al., 2022). However, most existing methods for this task are tailored for 2D images, limiting their effectiveness in 3D tasks. While some recent works explore UDA on 3D images (Shin et al., 2023; Xian et al., 2023), they typically convert 3D input into 2D slices for network processing, resulting in a loss of spatial information. More recently, MAPSeg (Zhang et al., 2024a) proposed a voxel-level pseudo-labeling method based on MAE. However, label distortion remains a main challenge for UDA.

In this paper, we introduce a novel UDA approach using large simulated macromolecular data (Eisenstein et al., 2019; Hagen et al., 2017) as the source domain dataset while experimental data as the target domain dataset. Advancements in data simulation techniques have facilitated the generation of cryo-ET subtomogram data beyond conventional biological methods. Given the structure of macromolecules, existing generative methods (Harar et al., 2023; Martinez-Sanchez et al., 2024) could create realistic synthetic datasets with corresponding voxel-level segmentation masks, offering a cost-effective alternative to traditional methods that required high-end equipment and significant human expertise. Nevertheless, the substantial differences between the two domains introduce new challenges for the UDA task. Firstly, simulated data is generated using fixed parameters, resulting in a consistent noise level across all subtomograms (typically 0.03 dB or 0.05 dB). In contrast, noise rates in experimental datasets are unpredictable. Examples of cryo-ET subtomograms are shown in Fig. 2. As shown in Fig. 2(a), both simulated and experimental subtomograms share the same voxel-space input representation. However, since the simulated data contain a consistent noise level, applying a fixed-range filter reveals the approximate particle shapes, as illustrated in Fig. 2(b). In contrast, the experimental data exhibit much more complex and heterogeneous noise, and even after applying the same fixed-range filter, no particle-related structural information can be clearly observed (Fig. 2(d)). Secondly, although subtomogram segmentation is inherently a binary task, the molecular categories present in the simulated subtomograms often differ from those in experimental data. This discrepancy might bias the segmentation model toward simulated data, leading to a domain shift problem.

To address the aforementioned challenges, we propose a Voxel-wise UDA framework, termed Vox-UDA, for cryo-ET subtomogram segmentation. Vox-UDA comprises two key components: a noise generation module (NGM) and a denoised pseudo-labeling (DPL) strategy. NGM generates Gaussian noise from a subset of the target dataset and applies it to the source samples, producing a target-like noisy effect. Meanwhile, DPL introduces an improved bilateral filter that replaces pixel differences with gradient differences, enhancing its effectiveness for 3D gray-scale images. Additionally, DPL preserves edge information during denoising as much as possible, resulting in undistorted pseudo-labels. These pseudo-labels provide additional supervision signals to mitigate the domain shift problem, thereby enhancing the model’s performance on the target data.

Although Vox-UDA (originally proposed in the conference version (Li et al., 2025a)) effectively eliminates the reliance on labeled experimental data and addressed the issue of domain shift problem caused by particle difference through simulating the noise environment and pseudo-labeling, it still relies on a certain amount of unlabeled experimental data (target dataset) for noise and pseudo-label generation. However, in real-world scenarios, due to data scarcity, it is challenging to ensure that a sufficient amount of experimental data can be collected under all conditions. For example, as shown in some released dataset from EMPIAR (Iudin et al., 2016) platform, the subtomogram dataset obtained from common biological tissues typically contains thousands of samples (Guo et al., 2018; Pacheco-Fiallos et al., 2023), while in a significant portion of other datasets (such as disease samples), the number of available samples is often limited to just a few dozen or even fewer. Therefore, this raises a new challenge: • *is it possible to train a highly generalizable model using only the simulated data without relying on any experimental data?*

To tackle this issue, in this paper, we further consider a scenario that is more in line with the real world, where the experimental data (target dataset) are not accessible during training process, and we introduce a Voxel-wise Zero-shot Domain adaptation framework for cryo-ET subtomogram segmentation, termed Vox-ZSDA. Fig. 1 illustrates the difference between Vox-UDA (Li et al., 2025a) and Vox-ZSDA. In detail, Vox-ZSDA follows a dual network scheme and utilizes a self-supervised graph learning strategy (SGL) to enhance the model’s sensitivity to particle structures. SGL employs a Nodes Contrastive Learning (NCL) loss between the feature graph nodes output from the student and teacher model. Additionally, as aforementioned, the noise rates of the source and target data are different. To further mitigate the effect of interference, different from existing self-supervised learning approaches which uses random masking (Huang et al., 2023; Xie et al., 2022b) strategy for self-training, we present a new Dynamic Graph Contrastive Learning (DGCL) technique. DCS takes the prominent features of noise and molecular structures as clustering centers and then performs graph constructions based on K-Nearest Neighbors (KNN) algorithm. On other other hand, it further selectively applies NCL loss to the graph nodes based on the clustering centers, avoiding noise centers and choosing the edge/semantic center, compelling the model to focus on molecular structure features rather than the noise components. This comprehensive ZSDA framework addresses the challenges of improving the generalization of cryo-ET subtomogram segmentation while reducing the dependence on the target dataset.

In summary, our contributions are as follows:

- To the best of our knowledge, we are the first to establish a paradigm for voxel-wise UDA segmentation (termed Vox-UDA) and ZSDA segmentation (termed Vox-ZSDA) in cryo-ET images. Our approach eliminates the reliance on large amounts of labeled experimental data by transferring knowledge learned from zero-cost simulated data to the real ones, enabling segmentation on experimental cryo-ET subtomograms.
- We propose Vox-UDA, which incorporates a noise generation module (NGM) and a denoised pseudo-labeling (DPL) strategy to empower the simulation of a target-like noisy effect, and provides additional supervision signals to address domain shift.
- We further propose Vox-ZSDA to incorporate self-supervised graph learning (SGL) into source training to eliminate the training dependency on the target dataset.

SGL comprises a Nodes Contrastive Learning (NCL) loss and a Dynamic Graph Contrastive Learning (DGCL) technique, designed to direct the model’s attention toward molecular structure features, thereby enhancing its generalization ability.

- We provide the first benchmark for UDA and ZSDA in cryo-ET subtomogram segmentation across three experimental datasets, including two newly curated datasets that are publicly available for the first time.

## 2 Related Work

### 2.1 Domain Adaptation

#### 2.1.1 Unsupervised Domain Adaptation for Vision Tasks

Under the unsupervised domain adaptation (UDA) settings, there are two types of datasets being used for training: the source domain dataset which is fully labeled, and the target domain dataset, which is unlabeled. The first UDA approach, introduced by (Ganin et al., 2016), leverages adversarial learning to adapt a model trained on source data to target data without requiring additional annotations. Since UDA greatly expands the model’s generalization ability, it allows adaptation to new domains without the need for labeled data in the target domain, making it applicable to a wide range of tasks. As cryo-ET subtomogram segmentation is a segmentation task, in this paper, we mainly focus on the UDA approaches applied in semantic segmentation tasks. For segmentation, the UDA methods aim to eliminate the cross-domain discrepancies through the content at both the feature- (Zou et al., 2018) and pixel-level (Zheng et al., 2023d), and have achieved excellent results in medical image segmentation (Huang et al., 2025; Li et al., 2022; Zhang et al., 2023a). Although these methods achieve great performance in UDA segmentation, they are primarily designed for 2D images, which are not suitable for cryo-ET subtomogram segmentation as tomographies are often volumetric images. To handle the UDA challenge in 3D segmentation tasks, Shin et al. (2023) proposes a cross-modality translation method to generate synthetic 3D target volumes from source 2D scans. Xu et al. (2023a) applied a fast Fourier transform to convert input 2D slices into the frequency domain. A consistency loss was utilized to simultaneously constrain both the feature domain and frequency domain to achieve a better UDA outcome. In this work, we would focus on the noise discrepancies between simulated and experimental subtomogram data, aiming to improve the model’s robustness to noise.

#### 2.1.2 Zero-shot Domain Adaptation for Vision Tasks

Zero-shot domain adaptation (ZSDA) is a target-free special case of UDA, aiming to train the model using only the source data, with the target data being entirely inaccessible throughout the training process. This concept was first proposed by Peng et al. (2018), by introducing a task-irrelevant domain-pairs to simulate the source-target domain situation and learn the adaptation ability, and transfer this ability to the source domains to achieve model generalization in the target domain. Building on the introduction of irrelevant pairs, some studies have enhanced ZSDA performance by incorporating semantic information (Kumagai & Iwata, 2018; Zhang et al., 2023b), feature disentanglement (Jhoo & Heo, 2021) or employing feature alignment techniques (Liu et al., 2024; Yang et al., 2025). Another distinct approach involves using generative models to generate domain pairs (Azuma et al., 2024; Garbin et al., 2020; Li et al., 2024), thereby augmenting the training samples with synthetic data. Such method has achieved outstanding performance in certain specific scenarios, such as day-light domain adaptation (Du et al., 2024; Lengyel et al., 2021; Luo et al., 2023). Recent studies on vision-language models have even inspired new ideas. P∅DA (Fahes et al., 2023) enhances the model’s inference ability on unseen target data by leveraging differences in text prompts and contrastive learning. ULDA (Yang et al., 2024) further proposes an unified framework, which no longer relies on domain-specific customization requirements for prompting. In this research direction, existing approaches mainly focus on images from nature or some specific real-world scenarios, such as road traffic, leaving the investigation on biological and 3D voxel-level data remain unexplored. In cryo-ET subtomogram segmentation, however, the domain gap primarily arises from differences in imaging conditions, leading to distinct noise characteristics across datasets. Such noise-related discrepancies cannot be explicitly captured or represented through language prompts. Therefore, unlike language-guided ZSDA methods, our framework does not rely on textual guidance but instead focuses on simulating and adapting to target-specific noise distributions. To address this challenge, we draw inspiration from graph contrastive learning (Xia et al., 2022a; You et al., 2020) and propose a self-supervised graph learning strategy that enables the model to focus on macromolecular structures and better adapt to cryo-ET subtomogram inputs from different domains.

### 2.2 Cryo-Electron Tomography

Cryo-electron tomography (cryo-ET) integrates cryogenic specimen preparation, electron microscopy for data acquisition, and tomographic reconstruction for 3D visualization (Koning, 2010). This technique allows biologists to capture structural information of macromolecules in an ultra-low temperature environment, which holds significant importance in the fields of biology and medicine. A cryo-ET subtomogram is a small cubic sub-volume extracted from a tomogram, normally only a single macromolecular complex is contained in each subtomogram. Deep learning based applications on cryo-ET have drawn widespread interest because of their potential to aid in the corresponding cryo-ET tasks, *e.g.,* subtomogram alignment (Zeng & Xu, 2020), subtomogram classification (Bandyopadhyay et al., 2022; Wan et al., 2024) and subtomogram segmentation (Zhu et al., 2021). However, deep learning methods rely on large amounts of data annotation, which is particularly challenging for cryo-ET images. Bandyopadhyay *et al.* proposed a domain randomization-based approach to enhance the generalization performance of the model in subtomogram classification (Bandyopadhyay et al., 2022). Zhu *et al.* proposed a weakly supervised approach, which used only 2D-level annotation for voxel segmentation to alleviate the burden of annotation (Zhu et al., 2021). In this paper, we would propose an UDA and a ZSDA approach for subtomogram segmentation, which together aim at utilizing a large amount of cost-free annotated simulated data for knowledge transfer, enabling the segmentation network to generalize on experimental cryo-ET subtomograms.

### 2.3 Graph Learning in Computer Vision

Recent studies on graph learning have proven their strong ability in constructing image structures in non-Euclidean space. Graph Neural Network (GNN) was first used as a fully connected network for image segmentation (Chandra et al., 2017; Krähenbühl & Koltun, 2011), which aimed to build relationships among all the pixel-pairs. Graph Convolutional Network (GCN) was further proposed to update feature embeddings within the visual graphs (Gao et al., 2019; Kazi et al., 2022). To alleviate the training burden caused by the fully-connected graph, dynamic graph learning has been proposed, aiming to selectively construct visual graphs based on the pixel or feature embeddings (Hu et al., 2020; Zhang et al., 2023c). Visual graphs have been applied to varies natural scenarios (Jia et al., 2024; Wang et al., 2024) and biomedical scenarios (Cen et al., 2024; Khanna et al., 2023). While most researches focus on 2D imaging, some studies have also started exploring graph construction for 3D scenes. EV-VGCNN (Deng et al., 2022) introduced a light GCN framework based on the sparsity of graph representations, and SVGA-Net (He et al., 2022) proposed a sparse graph attention network in point cloud space for 3D object detection. Unlike existing graph learning methods, we adopt a self-supervised learning strategy with a dynamic graph contrastive learning technique, where graphs are constructed using node centers extracted from noise filters. The contrastive learning process is implemented through different construction strategies.

## 3 Voxel-wise Unsupervised Domain Adaptation

Our proposed Vox-UDA is based on VoxResNet (Chen et al., 2018), a state-of-the-art method designed for full-ysupervised voxel-level segmentation. Given a source domain dataset $𝒮={\left\{x_{i}^{s},y_{i}^{s}\right\}}_{i=1}^{N}$ and a target domain dataset $𝒯={\left\{x_{j}^{t}\right\}}_{j=1}^{M}$, where $x_{i}$ represents the input 3D subtomogram and $y_{i}$ denotes the 3D ground-truth mask, we strive to train a voxel segmentation network for the target domain solely by leveraging ground-truth supervision signals from the source domain. Fig. 3 illustrates the details of the proposed Vox-UDA. As can be seen from the illustration, Vox-UDA takes $x_{i}^{s}$, $x_{i}^{t}$ and a subset of 𝒯 as input. This subset ${𝒯}_{N_{\text{sampled}}}$ is randomly sampled from 𝒯, which contains $N_{\text{sampled}}$ samples. ${𝒯}_{N_{\text{sampled}}}$ is then sent to the noise generation module (NGM) to obtain the target-like voxel-wise Gaussian noise $\epsilon ∼𝒩\left(0,\sigma_{t}^{2}I\right)$. Further, ϵ is introduced to the source input $x_{i}^{s}$ to produce updated input $x_{i}^{s^{\prime }}$. $x_{i}^{s}$, $x_{i}^{s^{\prime }}$ and $x_{j}^{t}$ are all passed to the student network to acquire segmentation loss ${ℒ}_{\text{seg}}$, consistency loss ${ℒ}_{\text{con}}$ and discriminator loss ${ℒ}_{\text{dis}}$ for optimization. Hence, the overall loss can be rewritten as

(1) $$ℒ={ℒ}_{\text{seg}}+{ℒ}_{\text{con}}+{ℒ}_{\text{dis}}.$$

Furthermore, to address the domain shift problem, we propose a denoised pseudo-labeling strategy. Specifically, $x_{j}^{t}$ is first processed using the improved bilateral filter (IBF) to remove noise before being fed into the teacher network to generate a pseudo-label. This pseudo-label is then utilized to refine the student network for enhanced performance. Notably, the threshold *η* for pseudo-label selection is set to 0.85, and the teacher network is updated using an exponential moving average (EMA).

### 3.1 Noise Generation Module

Given a sample $x_{n}^{t}$ from the input ${𝒯}_{N_{\text{sampled}}}$, we first apply Discrete Fourier Transform (DFT) to obtain its frequency information

(2) $${\overset{ˆ}{x}}_{n}(u,v,\zeta )=\xi \left[x^{t}\right],$$

where u,v and ζ represent the spatial frequencies of the Fourier transform, and *ξ* denotes the Discrete Fourier transform. In the frequency domain, low-frequency information primarily captures the textural details of the target object, whereas high-frequency information typically contains substantial noise with minimal edge details related to the object. To obtain the noise encompassed in the high-frequency information, $x_{n}(u,v,\zeta )$ is then passed to a high-pass filter to eliminate the textural details contained in low-frequency information

(3) $${\overset{ˆ}{x}}_{n}^{\prime }(u,v,\zeta )=H_{\text{high}}(u,v,\zeta ){\overset{ˆ}{x}}_{n}(u,v,\zeta ),$$

where $H_{\text{high}}(u,v,\zeta )$ denotes the high-pass filter. The filter rate is set to 24.4% (see more discussions in Sec. 5.3.1), which means only 24.4% remains while the rest of them are filtered. Inverse Discrete Fourier transform (iDFT) is further applied to recover voxel-level information from the filtered frequency domain $x_{n}^{\prime }(u,v,\zeta )$:

(4) $$x_{n}^{t^{\prime }}=\xi^{-1}\left[{\overset{ˆ}{x}}_{n}^{\prime }\right],$$

Where $\xi^{-1}[\cdot ]$ denotes the Inverse Discrete Fourier Transform. As mentioned in Sec. 1, the noise level of each input from the target domain is unpredictable. Therefore, instead of relying on the noise from a single $x_{n}^{t}$ , we compute the average noise level ${\overset{‾}{x}}_{n}^{t^{\prime }}$ across the entire subset for a more stable estimation:

(5) $${\bar{x}}_{n}^{t^{\prime }}=\frac{1}{N_{\text{sampled}}}\overset{N_{\text{sampled}}}{\underset{n=1}{\sum }}x_{n}^{t}$$

Since deep learning models are more sensitive to noise that conforms to a probability distribution (Lehtinen et al., 2018), we set the Gaussian noise as the input noise for noise generation (see detailed discussions in Sec. 5.3). Therefore, instead of directly introducing ${\bar{x}}_{n}^{t^{\prime }}$ to the source input $x_{i}^{s}$ , we only take its variance $\sigma_{t}^{2}$ and generate a Gaussian noise based on

(6) $$\epsilon =𝒩\left(0,\sigma_{t}^{2}I\right),\sigma_{t}^{2}=\frac{1}{N_{\text{sampled}}}\overset{N_{\text{sampled}}}{\underset{n=1}{\sum }}{\left({\bar{x}}_{n}^{t^{\prime }}-x_{n}^{t}\right)}^{2}$$

where $𝒩\left(0,\sigma_{t}^{2}I\right)$ denotes a random generated Gaussian noise with expectation equals to 0, and variance equals to $\sigma_{t}^{2}$. Meanwhile, the updated source input is obtained through

(7) $$x_{i}^{s^{\prime }}=x_{i}^{s}+\epsilon .$$

$x_{i}^{s}$ and $x_{i}^{s^{\prime }}$ are both sent to the student network to obtain the consistency loss ${ℒ}_{\text{con}}$. Following VoxResNet, we also take the output feature embeddings from the same layers as the input of the loss function

(8) $${ℒ}_{con}=\lambda_{1}{ℒ}_{BN}\left(f_{c},f_{c}^{'}\right)+\lambda_{2}{ℒ}_{BN}\left(f_{v2},f_{v2}^{'}\right)+\lambda_{3}{ℒ}_{BN}\left(f_{v4},f_{v4}^{'}\right)+\lambda_{4}{ℒ}_{BN}\left(f_{v6},f_{v6}^{'}\right),$$

where *ℒ_BN_* denotes the cosine similarity loss and $\lambda_{n}$ denotes the weights to control the relative importance among different consistency losses. Although NGM is introduced to simulate target-like noises, it is impossible to replicate a noise environment in the source domain that is entirely identical to the target domain. On the other hand, the shallower layers of the decoder primarily capture textural information, whereas the deeper layers focus more on edge details (Chen et al., 2017). To overcome these constraints, rather than applying an equal superposition, we assign different weights to different layers to balance the contributions of texture and edge consistency losses effectively.

### 3.2 Denoised Pseudo-Labeling

Although the NGM can narrow the noise level disparities between the two domains, as aforementioned, the segmentation network remains biased toward the source data due to the domain shift problem. To mitigate this issue, we may introduce an additional supervision signal through pseudolabeling. However, since the noise level in the target domain is unknown and can potentially distort the pseudo-labels, leading to degraded model performance, we specially propose a denoised pseudo-labeling strategy to enhance the reliability and robustness. Unlike the existing pseudo-labeling method whereby adding an extra training step, we tactfully use the student-teacher structure (Sohn et al., 2020). Before $x_{j}^{t}$ is sent to the teacher network to obtain the pseudo-label, we first perform denoising on $x_{j}^{t}$. We designed three different denoising methods: 1) directly using NGM for denoising, 2) using bilateral filter for denoising, and 3) using our designed improved bilateral filter (IBF) for noise reduction Fig. 4.

#### NGM Denoising.

The $x_{j}^{t}$ is directly sent to the NGM to obtain its noise $x_{j}^{t^{\prime }}$. Hence, the denoised image can be represented as ${\tilde{x}}_{j}^{t}=\left(x_{j}^{t}-x_{j}^{t^{\prime }}\right)$.

#### Bilateral Filter Denoising.

Although noise can be partially removed through frequency domain analysis, some edge information is inevitably lost in the process, leading to distortion of the pseudo-labels. Therefore, we further deploy a non-linear approach, *i.e.,*ilateral filter (Elad, 2002), as the denoiser instead of the NGM. bilateral filter (BF) consists of a domain Gaussian kernel and a range Gaussian kernel, the former is used to eliminate the noises, and the latter is to retain edge information as much as possible during filtering. BF uses a sliding window, extracting a 3 × 3 × 3 sub-figure for filtering operations each time. Given the central voxel $v_{p}$ and the rest of voxels $v_{p}$*, q∈V* of the sub-figure, the updated voxel can be represented as

(9) $$v_{q}^{\prime }=\frac{\sum_{q\in V}G_{\sigma_{d}}(\|p-q\|)G_{\sigma_{r}}\left(v_{q}-v_{p}\right)\times v_{q}}{\sum_{q\in V}G_{\sigma_{d}}(\|p-q\|)G_{\sigma_{r}}\left(v_{q}-v_{p}\right)},$$

where ‖⋅‖ denotes the Euclidean distance (Wang et al., 2005), $G_{\sigma }$ denotes the general form of the Gaussian kernel

(10) $$G_{\sigma }(x)=\frac{e^{-\frac{x^{2}}{2\sigma^{2}}}}{2\pi \sigma },$$

$\sigma_{d}$ and $\sigma_{r}$ denote the domain hyperparameter and range hyperparameter. Noted that both $G_{\sigma_{d}}(\cdot )$ and $G_{\sigma_{r}}(\cdot )$ follow the same Gaussian form as in Eq. 10, differing only is their standard deviation. Hence, the denoised image can be represented as ${\tilde{x}}_{j}^{t}=BF\left(x_{j}^{t}\right)$.

#### IBF Denoising.

The key point of the bilateral filter is the design that using two separate Gaussian kernels for different tasks. However, the range kernel introduced for retaining edge information mainly focuses on the voxel-level color difference, which indeed can achieve satisfactory results in the RGB space, but in the grayscale space, there might be more differences in brightness, which could affect the effectiveness of this kernel. Therefore, we further propose an improved bilateral filter (IBF), which uses the gradient of each voxel instead of its value for the range kernel for edge retaining. In detail, we use the 3D Sobel operator (Zhang, 1993) to calculate the gradient of each voxel $v_{q}$ in the *h*, *w*, and *d* (height, width, and depth) directions in three-dimensional space. Since voxel space is discrete, the gradient of $v_{q}$ in each direction can be represented as

(11) $$\frac{\partial \Delta }{\partial {\vec{v}}_{q}^{h}}=\Delta \left(v_{q+1}^{h},v_{q}^{w},v_{q}^{d}\right)-\Delta \left(v_{q-1}^{h},v_{q}^{w},v_{q}^{d}\right),$$

(12) $$\frac{\partial \Delta }{\partial {\vec{v}}_{q}^{w}}=\Delta \left(v_{q}^{h},v_{q+1}^{w},v_{q}^{d}\right)-\Delta \left(v_{q}^{h},v_{q-1}^{w},v_{q}^{d}\right),$$

(13) $$\frac{\partial \Delta }{\partial {\vec{v}}_{q}^{d}}=\Delta \left(v_{q}^{h},v_{q}^{w},v_{q+1}^{d}\right)-\Delta \left(v_{q}^{h},v_{q}^{w},v_{q-1}^{d}\right),$$

where $x_{q}^{h},x_{q}^{w}$ and $x_{q}^{d}$ denote the values of $v_{q}$ in the *h*, *w*, and *d* directions, and Δ denotes the Sobel operator. Moreover, compared to the gradient of the central voxel $v_{p}$, if a voxel belongs to the object (*i.e.,* is inside), its gradient should be similar to that of $v_{p}$. Otherwise, the difference between the two gradient should be significant. In other words, we can replace the second filter in Eq. 9 and obtain the improved bilateral filter (IBF(·)):

(14) $$v_{q}^{\prime }=\frac{\sum_{q\in V}G_{\sigma_{d}}(\|p-q\|)G_{\sigma_{r}}\left(\frac{\partial \Delta }{\partial {\vec{v}}_{p}}-\frac{\partial \Delta }{\partial {\vec{v}}_{q}}\right)\times v_{q}}{\sum_{q\in V}G_{\sigma_{d}}(\|p-q\|)G_{\sigma_{r}}\left(\frac{\partial \Delta }{\partial {\vec{v}}_{p}}-\frac{\partial \Delta }{\partial {\vec{v}}_{q}}\right)},$$

where

(15) $$\frac{\partial \Delta }{\partial {\vec{v}}_{p}}-\frac{\partial \Delta }{\partial {\vec{v}}_{q}}=\left(\frac{\partial \Delta }{\partial {\vec{v}}_{p}^{h}},\frac{\partial \Delta }{\partial {\vec{v}}_{p}^{w}},\frac{\partial \Delta }{\partial {\vec{v}}_{p}^{d}}\right)-\left(\frac{\partial \Delta }{\partial {\vec{v}}_{q}^{h}},\frac{\partial \Delta }{\partial {\vec{v}}_{q}^{w}},\frac{\partial \Delta }{\partial {\vec{v}}_{q}^{d}}\right).$$

Consequently, the denoised image is denoted as ${\tilde{x}}_{j}^{t}=$ $IBF\left(x_{j}^{t}\right).{\tilde{x}}_{j}^{t}$ is further sent to the teacher network and obtain the pseudo-label with the threshold *η*. The pseudo-label is further sent back to the student network as a supervision signal for the target flow.

## 4 Voxel-wise Zero-shot Domain Adaptation

### 4.1 Limitations of Vox-UDA

Vox-UDA addresses the the high training costs caused by the strong dependence of existing methods on annotated data. However, as can be seen from Sec. 3.1, the noise generation process still requires a large amount of target data. In some real-world scenarios, it is not possible to obtain a large amount of target data in advance, such as in fast automatic particle picking (Rath & Frank, 2004; Tegunov & Cramer, 2019) or the collection of certain rare molecular types. Therefore, we further explore the potential of zero-shot domain adaptation and propose Vox-ZSDA that eliminates the need for experimental data. Vox-ZSDA consists of a self-supervised graph learning (SGL) strategy and a dynamic graph contrastive learning (DGCL) technique.

### 4.2 Clarification of UDA, DG, and ZSDA

Before introducing the details of the proposed Vox-ZSDA, we briefly clarify the distinction between Unsupervised Domain Adaptation (UDA), Domain Generalization (DG), and Zero-Shot Domain Adaptation (ZSDA) in the context of cryo-ET segmentation. Given a labeled source domain dataset *𝒮* and an unlabeled target domain dataset *𝒯*, UDA aims to train a model using both *𝒮* and *𝒯*, while supervision is provided only by labels from *𝒮*. DG, in contrast, trains a model solely on *𝒮* and evaluates it directly on *𝒯*. In both UDA and DG, the source and target domains are assumed to share the same label space, i.e., ${𝒴}_{s}={𝒴}_{t}$. ZSDA relaxes the closed-set assumption by allowing ${𝒴}_{s}\neq {𝒴}_{t}$,and in particular ${𝒴}_{t}\setminus {𝒴}_{s}\neq \emptyset$, which implies that the model must generalize to previously unseen labels at test time without accessing any target-domain supervision.

### 4.3 Self-supervised Graph Learning

Following Sec. 3, we set the same pair of Siamese networks, and denote the parameters of student and teacher network by ${𝒲}^{s}$ and ${𝒲}^{t}$. We also employ the VoxResNet as the basic network. Different from Vox-UDA, only the source domain dataset $𝒮={\left\{x_{i}^{s},y_{i}^{s}\right\}}_{i=1}^{N}$ is used for Vox-ZSDA training. As can be seen from Fig. 5, the input subtomogram is sent to both student and teacher network. Due to the differences in noise and molecular structure between the target data and the source data, we aim to train a model that focuses more on texture and boundary details rather than the overall 3D image. Hence, we apply a self-supervised learning (SGL) strategy based on graph learning from shallow to deep layers. As there are six VoxReS blocks in each model, we take the 1*_st_*, 3*_rd_* and 5*_th_* blocks (note, using 2*_rd_*, 4*_th_* and 6*_th_* is equally valid and does not impact the results) to perform SGL. Given the output feature embeddings from these blocks

(16) $$F_{n}^{s}=S\left({𝒲}_{n}^{s};x_{i}^{s}\right),F_{n}^{t}=T\left({𝒲}_{n}^{t};x_{i}^{s}\right),n=\{1,3,5\},$$

where $F_{n}^{s}$ and $F_{n}^{t}$ denote the output features from the student and teacher model, and $S\left({𝒲}_{n}^{s};\right),T\left({𝒲}_{n}^{t};\right)$ denote the $n_{th}$ VoxReS block. $F_{n}^{s}$ and $F_{n}^{t}$ are further sent to SGL for graph construction.

#### 4.3.1 Graph Construction for Student Network

Normally, there are two ways to perform graph projection for visual tasks: pixels/feature points as nodes (Li & Gupta, 2018; Li et al., 2023; Zhai et al., 2021) or K-Nearest Neighbor (KNN) (Wang et al., 2012; Yao et al., 2024). To support the subsequent DGCL module, in this paper, we use the KNN method for graph construction. Given the input feature $F_{n}^{s}\in R^{C\times H\times W\times D}$ from student network, $F_{n}^{s}$ is first projected into graph vectors through the clustering centers $C=\left\{c_{0},\cdots ,c_{j}\right\}$ and local bandwidth $\sigma_{j}$:

(17) $$g_{j}=\frac{\sum_{k}q_{k}\times f_{k}^{s}}{\sum_{k}q_{k}},$$

where $g_{j}$ denotes the node within graph ${𝒱}^{s}\in R^{C\times j}.f_{k}^{s}$ is obtained throug KNN:

(18) $$f_{k}^{s}\in N_{k}\left(f_{hwd}^{s}\right)=\{f_{hwd}^{s}|h,w,d\in \text{arg}\;\underset{h,w,d}{\text{min}\;}\text{cos}(\theta ),\text{and}\left|N_{k}\left(f_{hwd}^{s}\right)\right|=k\},$$

where $f_{hwd}^{s}\in R^{C\times 1}$ denotes the feature vector of $F_{n}^{s}$ at point (h,w,d),cos(θ) denotes the cosine similarity between $f_{hwd}^{S}$ and the center $c_{j}$, and $q_{k}$ denotes the soft assignment calculated through softmax matrix

(19) $$q_{k}=\frac{\text{exp}\left(-{\left\|f_{k}^{S}-c_{j}\right\|}^{2}/2\sigma_{j}^{2}\right)}{\sum_{k}\text{exp}\left(-{\left\|f_{k}^{S}-c_{j}\right\|}^{2}/2\sigma_{j}^{2}\right)}.$$

Different from Li and Gupta (2018); Zhai et al. (2021), which set the global bandwidth as a learnable parameter that is not suitable for high-dimensional data and non-uniformly distributed data, we compute the local bandwidth $\sigma_{j}$ based on the *k* nearest feature vectors

(20) $$\sigma_{j}=\frac{1}{k}\underset{k}{\sum }\left\|f_{k}-c_{j}\right\|,$$

allowing it to automatically adapt to data density. We further calculate the graph adjacent matrix ${𝒜}^{s}={𝒱}^{s⊤}\times {𝒱}^{s}$

#### 4.3.2 Graph Construction for Teacher Network

Given the input feature $F_{n}^{t}\in R^{C\times H\times W\times D}$ from teacher network, similar to student network, the graph projection process follows the same equations as Eq. (17),(18),(19) and (20). Because our self-supervised learning is trained using a multi-view graph-level contrastive learning approach, unlike Sec. 4.3.1, which employs cosine similarity for graph construction, we utilize Chebyshev Distance (Coghetto, 2016; Shieh & Tsai, 2010) instead, in the teacher network (see detailed discussion on the choice of distance metrics in Sec. 5.3.2). Hence, the Eq. (18) is replaced by

(21) $$\begin{array}{l} f_{k}^{t}\in N_{k}\left(f_{hwd}^{t}\right)=\\ \{f_{hwd}^{t}|h,w,d\in \text{arg}\;\underset{h,w,d}{\text{min}\;}d_{c}\left(f_{hwd}^{t},c_{j}\right),\\ and\;|N_{k}\left(f_{hwd}^{t}\right)|=k\}, \end{array}$$

where $d_{c}(\cdot )$ denotes the Chebyshev Distance

(22) $$d_{c}\left(f_{hwd}^{t},c_{j}\right)=\underset{m}{max}\left|{f_{hwd}^{t}}^{\left(m\right)}-c_{j}^{\left(m\right)}\right|,$$

and *(m)* denotes the $m_{th}$ dimensional feature. Herein the graph and graph adjacent matrix of the teacher network can be formulated as ${𝒱}_{n}^{t}\in R^{C\times j}$ and ${𝒜}^{t}={𝒱}^{t^{⊤}}\times {𝒱}^{t}$.

### 4.4 Dynamic Graph Contrastive Learning

To provide additional supervision and encourage the model to focus on molecular structure, we introduce the Nodes Contrastive Learning (NCL) loss. Specifically, we select nodes corresponding to the same cluster center in the two graphs ${𝒱}_{n}^{s}=\left\{{𝒱}_{1}^{s},{𝒱}_{3}^{s},{𝒱}_{5}^{s}\right\}$ and ${𝒱}_{n}^{t}=\left\{{𝒱}_{1}^{t},{𝒱}_{3}^{t},{𝒱}_{5}^{t}\right\}$, and compute the contrastive loss between them

(23) $${ℒ}_{NCL}=-log\frac{\text{exp}\left(v_{i}^{s⊤}\times v_{i}^{t}\right)}{\sum_{j}\text{exp}\left(v_{i}^{s⊤}\times v_{j}^{t}\right)},$$

where $v_{i}^{s}$ and $v_{i}^{t}$ denote nodes from ${𝒱}_{n}^{s}$ and ${𝒱}_{n}^{t}$ , respectively. In practice, we perform L2 normalization on the node embeddings and use their inner product as the similarity measure, which constrains the value range to [−1, 1] and ensures stable optimization without additional scaling. Therefore, the temperature parameter is not included. As mentioned in Sec. 1, due to the differences in noise and structure between the source and target data, we aim for the model to focus more on the molecular structure itself. Hence, instead of using learnable clustering centers *C*, we utilize the IBF module proposed in Sec. 3.2 to dynamically construct clustering centers based on data noise, internal texture, and boundary information. Given the central voxel $v_{p}$ of the sliding window used in IBF, we first use the IBF in Eq. 14 to obtain the noise and transform it to have the same shape as $f_{hwd}^{s}\in R^{C\times 1}$:

(24) $$c_{\text{noise}}=conv\left(v_{q}-IBF\left(v_{q}\right)\right),$$

where $c_{\text{noise}}\in R^{C\times 1}$ denotes the noise-cluster center, and *conv*(·) denotes a 1 × 1 convolution with weights as an identity transformation. Similarly, the internal texture and boundary center can be formulated as

(25) $$c_{\text{edge}}/c_{\text{texture}}=conv\left(IBF\left(v_{q}\right)\right).$$

For better understanding, we visualize representative z-slices (Fig. 6(a–b)) of the feature maps in Fig. 6. The improved bilateral filter performs gradient-aware local aggregation, which suppresses high-frequency noise while preserving structural boundaries and textured regions. As a result, its responses naturally exhibit distinct patterns corresponding to noise-dominated and edge/texture-dominated components. The two heatmaps (Fig. 6(c–d)) respectively correspond to the noise and edge/texture clusters derived from the IBF-based decomposition, illustrating distinct spatial activation patterns across the subtomogram. We employ different domain and range Gaussian kernels to selectively capture noise, boundary and internal texture information for clustering centers *C*. In detail, we gradually set $\sigma_{d}$ to obtain more noise, and gradually lower $\sigma_{d}$ and increase $\sigma_{r}$ to maintain more edge information, while we gradually increase $\sigma_{d}$ and lower $\sigma_{r}$ to maintain more texture information. Every adjustment of increase and lower is made at a fixed interval determined by the hyperparameter ϕ. Each operation is repeated $\frac{j}{3}$ times to acquire multiple clustering centers. Therefore, the clustering centers can be represented as $C=C_{\text{noise}}\cup C_{\text{edge}}\cup C_{\text{texture}}$. In order to facilitate the model to selectively focus on texture or boundaries, we compute ${ℒ}_{NCL}$ for nodes corresponding to different centers in different VoxRes blocks, *i.e.,* for the 1*_st_* and 3*_rd_* block, the loss is only applied to *C_texture_*, and for 5*_th_* block, the loss is only applied to *C_edge_*. Since the molecular types of the target dataset may not necessarily be the same as those of the source, we assign a lower weight to the loss for the texture part. Therefore, the overall objective function of the student network can be expressed as

(26) $$ℒ=0.5*{ℒ}_{NCL}^{1}+0.5*{ℒ}_{NCL}^{3}+{ℒ}_{NCL}^{5}+{ℒ}_{\text{seg}},$$

where

(27) $${ℒ}_{seg}={ℒ}_{CE}\left(y_{i}^{s},𝒮\left({𝒲}^{s};x_{i}^{s}\right)\right).$$

And the teacher network is updated through exponential moving average (EMA).

To further understand how the proposed DGCL facilitates generalization across unseen domains, we interpret our framework from the perspective of domain adaptation theory. According to the domain adaptation bound (Ben-David et al., 2006), the target generalization error $R_{x\in 𝒯}(h)$ can be bounded by the source error $R_{x\in 𝒮}(h)$, the domain divergence $d_{ℋ\Delta ℋ}\left(P_{s},P_{t}\right)$ and the joint optimal error *ς*:

(28) $$R_{x\in 𝒯}(h)\leq R_{x\in 𝒮}(h)+d_{ℋ\Delta ℋ}\left(P_{s},P_{t}\right)+\varsigma ,$$

where $R_{x\in 𝒮}(h)={ℒ}_{\text{seg}}(y,h(x))$ and *P* denotes the distribution space of the source and target samples. In the context of cryo-ET subtomogram segmentation, the domain divergence term mainly arises from two factors: (1) differences in noise characteristics between simulated and experimental data, and (2) variations in particle structures. The proposed DGCL mitigates this divergence by aligning texture-related nodes in shallow layers $\left(0.5*{ℒ}_{NCL}^{1}+0.5*{ℒ}_{NCL}^{3}\right)$ and edge-related nodes in deeper layers $\left({ℒ}_{NCL}^{5}\right)$. The former helps the model to suppress noise-dominated discrepancies, while the latter enhances its sensitivity to particle boundaries and structural consistency. Together, these hierarchical contrastive constraints effectively reduce the divergence between source and target feature distributions through

(29) $$\underset{h}{min}d_{ℋ\Delta ℋ}\left(P_{s},P_{t}\right)\Rightarrow R_{x\in 𝒮}(h)\approx R_{x\in 𝒯}(h),$$

thereby improving generalization in the absence of any target-domain data access.

## 5 Experiments

### 5.1 Experimental Settings

#### 5.1.1 Implementation Details and Hyperparameters

We utilize the VoxResNet as the base architecture for both Vox-UDA and Vox-ZSDA. The whole experiments are conducted on one single NVIDIA A100 Tensor Core GPU with 80GB memory. For the training process of Vox-UDA and Vox-ZSDA, we choose the Adam optimizer with an initial learning rate set to 1e-3 for optimization, and both models are trained for 300 epochs with batch size of 16. The learning rate is decayed by 90% every 100 epochs. For Vox-UDA, the hyperparameters of sampled number $N_{\text{sampled}}$ and filter rate *ρ* are empirically set to 10 and 24.4%, respectively. The domain hyperparameter $\sigma_{d}$ and range hyperparameter $\sigma_{r}$ defined in the improved bilateral filter are set to 120 and 1.2, respectively. For Vox-ZSDA, the hyperparameters center number *j* , number of neighbors *k*, and adjustment rate *φ* used for the IBF module are set to 24, 10 and 20%. Through sensitivity analysis, we find that the model performance remains stable across different datasets when setting *j* within [20, 30] and *φ* within [18%, 23%], which we recommend as default ranges for new domains (see details in Sec. 5.3.2).

#### 5.1.2 Datasets and Evaluation Metrics

We conduct experiments on two types of datasets: simulated dataset (source dataset) and experimental dataset (target dataset).

##### Simulated Dataset.

The simulated dataset used as the source dataset is generated following the same generation process as Zeng et al. (2023). We choose six representative macromolecule complexes in our simulated datasets and divide them into two groups as two separate source datasets (1bxn, 1f1b, and 1yg6; 2byu, 2h12, and 21db). For each macromolecule complex, we simulate it with two different noise levels, with SNR of 0.03 and 0.05, and each of them contains 500 samples. Following existing work (Liao et al., 2020; Zeng & Xu, 2020), all the input subtomogram are resized to 32^3^. The simulated dataset contains 6,000 samples in total (3,000 samples for each source dataset).

##### Experimental Dataset.

We use three experimental datasets as the target datasets: *Mycoplasma pneumoniae* (O’Reilly et al., 2020), Erwinia (Prichard et al., 2023) and PolyGA (Guo et al., 2018). *Mycoplasma pneumoniae* and Erwinia are newly collected and pre-processed from the public repository EMDB (Consortium, 2024)^1^. ***Mycoplasma pneumoniae*** contains 10 cryo-ET subtomograms of wild-type Mycoplasma pneumoniae cells. Each subtomogram has a shape of 200^3^. Due to the size of each input subtomogram is too large for training, we resize each subtomogram to 192^3^ and cut each one into multiple non-overlapping patches with size 32^3^, and use each patch as the input for the model. **Erwinia** consists of 10 cryo-ET subtomograms of wildtype Erwinia phage vB_EamM_RAY baseplate complexes. The size of each subtomogram ranges from 72^3^ to 84^3^. We resize each subtomogram to 64^3^ and cut each one into multiple non-overlapping patches with size 32^3^, and use each patch as the input for the model. Hence, there are 2160 samples in *Mycoplasma pneumoniae* dataset and 80 samples in the Erwinia dataset. **Poly-GA** is a public datasetconsisting of 1,033 subtomograms from three macromolecular complexes-ribosome, 26S proteasome, and TRiC. Each subtomogram is also re-scaled to size 32^3^.

As there is no class overlap between the simulated and experimental datasets, we directly set the simulated dataset as the source domain and the experimental dataset as the target domain for the zero-shot domain adaptation task.

For evaluation, the mean intersection of union (**mIoU**) and dice similarity coefficient (**Dice**) are employed to evaluate the segmentation performance.

#### 5.1.3 Baselines

For both tasks, we set a “w/o adaptation” setting and a “Fully Supervised” setting for comparison. The “w/o adaptation” setting is an original VoxResNet trained on the source dataset without adaptation. The “Fully Supervised” setting is a VoXResNet trained on the target datasets with all labels provided, as the upper bound.

For UDA in cryo-ET subtomogram segmentation, we implement the most relevant traditional and state-of-the-art UDA approaches for 2D image segmentation on our task, including the single discriminator-based (DANN (Ganin et al., 2016)), the image synthesizing based (PDAM (Liu et al., 2020)) and the self-training based (Zhao et al. (2024b)). We also include Cryo-Shift (Bandyopadhyay et al., 2022), an UDA approach designed for cryo-ET classification. As Cryo-Shift is not designed for segmentation, we replace the base model with VoxResNet to modify Cryo-Shift for segmentation task. In the meantime, we also include the five most recent approaches designed for volumetric images, including ASC (Xu et al., 2023a), LE-UDA (Zhao et al., 2022), DAR-Unet (Yao et al., 2022), DDSP (Zheng et al., 2024c) and MAPSeg (Zhang et al., 2024a). ASC and LE-UDA cut 3D images into 2D slices and apply UDA on 2D scenario, while DAR-Unet, DDSP and MAPSeg directly performs UDA in 3D voxel space.

For ZSDA in cryo-ET subtomogram segmentation, we compare our method with the most classic as well as the most recent comparable ZSDA methods applied in 2D scenes, *i.e.,* three task-transfer based (*i.e.,* ZSDA (Wang & Jiang, 2020), ZDDA-C (Kutbi et al., 2021), and Liu et al. (2024)), together with three prompting based (*i.e.,* P∅DA (Fahes et al., 2023), ULDA (Yang et al., 2024) and UPRE (Zhang et al., 2025)). Since it is improbable to find an irrelevant task pair similar to cryo-ET, for task-transfer methods, we directly migrated task pairs from medical images to cryo-ET subtomograms. In practice, we set the CT/MRI as the task-irrelevant dual-domain pairs and use the Cardiac CT-MRI Dataset (Zhang et al., 2024a) as the training set. For prompting based methods, as there are currently no multimodal pre-training framework comparable to CLIP or large-scale paired datasets for cryo-ET study, we have to employ the BioBERT (Lee et al., 2020) pre-trained on the biomedical corpus as the text encoder, and combine it directly with VoxResNet to approximate a prompt-based setup. We utilize prompts such as “*cryo-ET subtomogram of XYZ protein from an/a experimental/simulated dataset*” and pair them with the corresponding subtomogram as the input-pair for training. Noted that since P∅DA requires target-like prompts for training the PIN module, we designed two distinct types of text prompts corresponding to the simulated and experimental datasets. For each source input, two separate prompts were used to indicate its simulation or experimental context, allowing the model to learn prompt-conditioned feature modulation through the PIN layer.

Each model (including ours) is run for five times with different random seed, and the mean and standard deviation of evaluation metric are reported in Table 1, 2, 3, 4, 5, 6.In addition, paired t-tests are performed across runs to assess the statistical significance of the performance gains, and all reported improvements are statistically significant (*p* < 0.05).

### 5.2 Comparisons with State-of-the- Arts

#### 5.2.1 Voxel-wise Unsupervised Doma in Adaptation

We report the segmentation results on the Poly-GA dataset in Table 1 by using the [1bxn, 1f1b, and 1yg6] as the source dataset. As can be observed in this table, our approach outperforms all the state-of-the-art methods. Compared with “w/o adaptation”, DANN and PDAM indeed boost the model’s performance, however, the effect is not obvious if compared with our Vox-UDA (w IBF) (*i.e.,* PDAM achieves 55.1 in *Dice* while Vox-UDA (w IBF) achieves 65.9). In addition, compared with three recent UDA methods, our proposed Vox-UDA (w IBF) still excels on target subtomogram segmentation, which leads to significant improvements in both *mIoU*(*i.e.,*41.5 → 50.3) and *Dice* (*i.e.,* 60.0 → 65.9). We also report extra UDA setting results in Table 2 by using the other three macromolecules [2byu, 2h12, and 21db] as source datasets, by which our proposed method again achieves state-of-the-art performance over all the comparison approaches. Fig. 7 shows the visualization of the segmentation results on the Poly-GA dataset under setting I.

We report the segmentation results on the *Mycoplasma pneumoniae* dataset and Erwinia dataset in Table 3 following the same experimental settings. As can be seen, our Vox-UDA performs better over all SOTAs, *e.g., mIoU* increased by 4.2% on the setting I of *Mycoplasma pneumoniae* dataset, and *Dice* increased by 2.7% on the setting II of Erwinia dataset. We provide the visualization results on *Mycoplasma pneumoniae* dataset in Fig. 8.

#### 5.2.2 Voxel-wise Zero-shot Domain Adaptation

Table 4 and Table 5 report the quantitative results of our model and baseline approaches on Poly-GA dataset. For a fair comparison, all models use the same basic network VoxRes-Net for training. As can be seen, the Vox-ZSDA performs better than all baselines, *e.g., mIoU* increased by 11.4% and *Dice* increased by 6.3%. This is because self-supervised graph learning strategy designed in this paper facilitates the model to focus on more on the molecular structures instead of the irrelevant information maintained in the subtomograms. Compared with task-transfer based baselines, due to the scarcity of data, task-transfer methods are not suitable for cryo-ET data. As for the prompting-based method that modifies the data domain, it is not very effective since cryo-ET data primarily differ in fine details rather than overall domain characteristics. Fig. 9 shows the visualization results of Poly-GA dataset under experimental setting I.

Similar to Vox-UDA task, we can report the segmentation results on the *Mycoplasma pneumoniae* dataset and Erwinia dataset in Table 6 following the same experimental settings. The superior results of our method demonstrate the outstanding generalization ability of our method on inferring molecular structures in cryo-ET subtomograms. The qualitative comparisons between Vox-ZSDA and other baselines are provided in Fig. 10.

### 5.3 Ablation Study

In this section, we provide extensive ablation studies on the components and hyperparameter settings of both Vox-UDA and Vox-ZSDA to analyze the effectiveness of our proposed methods.

#### 5.3.1 Vox-UDA

##### Effectiveness of the Improved bilateral filter.

To validate the effectiveness of the proposed improved bilateral filter (IBF) for denoised pseudo-labeling, we conduct experiments using the three different denoisers respectively, and report the segmentation results in both Table 1 and Table 2. As can be seen from these tables, comparing the method of using NGM as a denoiser, employing bilateral filtering (BF) indeed brings a performance improvement (*i.e., mIoU* increased 1.2% in Table 1 and *Dice* increased 1.0% in Table 2). This is because BF can preserve some edge information while denoising, thereby avoiding pseudo-label distortion. However, as discussed in Sec. 3.2, the range kernel of BF is not suitable for grayscale inputs. Our proposed IBF addresses this drawback by using a Laplacian transform, which allows the range kernel to focus more on gradient changes in the voxel space rather than value changes. Therefore, our new model achieves the best performance via the proposed IBF (*i.e., mIoU* increased 2.4% in Table 1 and *Dice* increased 3.3% in Table 2). We further compared our proposed IBF with a recently proposed learnable bilateral filtering method (Guo et al., 2025), which introduces an adaptive bilateral filter (ABF) using learnable parameters to dynamically control the filtering process, enabling the bilateral filter to better adapt to the distribution gap between different image resolutions. As shown in Table 7, although ABF improves performance compared with the standard BF, it still performs worse than our proposed IBF.

##### Effectiveness of Different Proposed Modules.

Table 8 shows the evaluation of the effectiveness of each module in our method. For comparison, we build a baseline only using a single discriminator with VoxResNet, which shows the same model structure as DANN (Ganin et al., 2016). From Table 8, we observe that both “w/o NGM” and “w/o PL” achieve better performance than “Baseline”, which demonstrates the effectiveness of our proposed two modules in dealing with the challenges for UDA in subtomogram segmentation. In the meantime, compared with using these two modules only, Vox-UDA achieves a significant performance improvement (*i.e., mIoU* improved by 15.8% and *Dice* improved by 11.0%).

##### Noise choice.

As mentioned in Sec. 3.1, we choose Gaussian noise in NGM for noise generation. To demonstrate the rationality of our choice, we provide additional experiments in Fig. 11, using Poisson noise (Carlavan & Blanc-Féraud, 2011) and Speckle noise (Maity et al., 2015) as the added noise for NGM, respectively. Given the variance $\sigma_{t}^{2}$ obtained through the average noise level ${\overset{‾}{x}}_{n}^{t^{\prime }}$ of the subset, the Poisson noise can be represented as $\epsilon_{p}=\pi \left(\sigma_{t}\right)$ and the Speckle noise can be formulated as

(30) $$\epsilon_{s}=x_{i}^{s}\times 𝒩\left(0,\sigma_{t}^{2}I\right),$$

where $x_{i}^{s}$ denotes the input source image. As can be seen from the figure, compared with the other two noises, using Gaussian noise achieves the best performance, demonstrating its suitability for simulating the target noise distribution in our setting. It is worth noting that, although the Gaussian noise assumption simplifies the complex noise characteristics of cryo-ET data, it provides a stable and tractable simulation strategy for domain adaptation. We acknowledge that real cryo-ET noise can exhibit non-Gaussian and structured patterns, arising from Poisson statistics, contrast transfer function (CTF) modulation, and missing-wedge artifacts during tomographic reconstruction. In future work, we will further extend our framework to incorporate more physically grounded and CTF-aware noise modeling.

##### Hyperparameter Analysis.

We herein further evaluate the hyperparameters used in our approach. Following the main experiments, each model is run for five times with different random seed to evaluate the stability of the hyperparameters, and the mean and standard deviation of evaluation metric are reported. As shown in Table 9, we evaluate the sampled number $N_{\text{sample}}$, the high-pass filter rate ρ, the weight $\lambda_{n}$ for consistency losses, the domain hyperparameter $\sigma_{d}$ and range hyperparameter $\sigma_{r}$. $N_{\text{sample}}$ controls the number of sampled targets for noise generation. Since noise distribution in the target domain is uneven, we use random sampling instead of the full dataset. An improper $N_{\text{sample}}$ - either too large or too small - negatively impacts performance. As shown in Table 9, $N_{\text{sample}}=10$ yields the best results, improving mIoU by 18% over $N_{\text{sample}}=20$ and *Dice* by 11% over $N_{\text{sample}}=15$. Additionally, ρ serves as the filter rate, determining the retained information for further processing. As aforementioned, noise primarily resides in the high-frequency components of 2D and 3D images. However, determining the balance between high and low frequencies in a high-pass filter is often subjective. To optimize this, we experiment with different retention levels of high-frequency information, finding that ρ=24.4% yields the best results, improving *mIoU* by 16% over ρ=17.8% and *Dice* by 10% over ρ=8.4%. As mentioned in Sec. 3.1 that for segmentation tasks, high-level features emphasize edges, while low-level features capture textures. Thus, we assign equal weights to $\lambda_{1}$ and $\lambda_{2}$ for low-level features and $\lambda_{3}$ and $\lambda_{4}$ for high-level ones, *i.e.,* $\lambda_{1}=0.2,\lambda_{2}=0.2,\lambda_{3}=0.3,\lambda_{4}=0.3$ achieves the best performance and brings a 19%mIoU and 14% Dice improvement. Lastly, we evaluate $\sigma_{d}$ and $\sigma_{r}$, confirming that our chosen values are optimal, as adjustments do not enhance model performance.

##### Feature Visualizations.

As shown in Fig. 12, we visualize the feature embeddings learned by the segmentation network with the commonly used t-SNE (Maaten & Hinton, 2008) method. Fig. 12(a) and (b) represent the visualization results of “after adaptation” and “before adaptation”, respectively. As can be observed from the figure, the distribution of the source and target features learned from our proposed method is more consistent compared to the distribution without adaptation. This indicates that our method has achieved knowledge transfer and generalized the model to the target data.

#### 5.3.2 Vox-ZSDA

##### Distance Metrics choice for Graph Projection.

As discussed in Sec. 4.3.2, we set the Chebyshev distance as the graph construction metric for the teacher network. This choice is motivated by the use of cosine similarity in the student network. While the teacher network necessitates a different graph construction approach, it should still be guided by the similarity between nodes and clustering centers rather than a direct computation of their value differences. We provide additional ablation study results by replacing the Chebyshev distance $\ell_{\infty }$ with the Euclidean distance $\ell_{2}$ in Table 10. As can be seen from the table, using $\ell_{2}$ for graph construction can achieve certain effectiveness; however, due to its distinct measurement approach compared to cosine similarity ($\left(i.e.,\ell_{2}\right.$ measures absolute magnitude differences, while cosine similarity quantifies directional differences) ), it hinders model convergence, ultimately leading to lower performance.

Beyond the choice of distance metrics, we also note that the scalability of graph construction is another practical consideration. Although KNN with cosine similarity works well in our current experiments, computational scalability issues may arise since the cryo-ET subtomograms used are relatively small (32^3^ voxels). For high-resolution 3D volumes, this could become a bottleneck. To address this, we are considering two promising directions: (1) replacing the global KNN graph with a dynamic local graph that updates neighborhood connections adaptively during training, and (2) adopting lightweight approximate nearest neighbor search algorithms to achieve more efficient and scalable graph construction. We plan to explore these directions in future work to further enhance the scalability and general applicability of our framework.

##### Graph Contrastive Learning Strategy.

To prove the effectiveness of the proposed Graph Contrastive Learning strategy, we additionally set up a randomly selected nodes setting for an ablation study on the contrastive learning strategy. The “Random” setting follows the same nodes number and the NCL loss *ℒ_NCL_* as our proposed strategy, except at each VoxRes block, it randomly selects the nodes for contrastive learning. For fair comparison, we repeat the experiments five times with different random selected nodes and choose the highest results. As can be seen from Table 10, due to the “Random” setting may introduces noise into contrastive learning, causing the model to be affected by the noise in the simulated data, which in turn biases it toward the source dataset.

##### Hyperparameter Analysis.

Similar to the ablation analysis of Vox-UDA, we also evaluate the hyperparameters in the proposed Vox-ZSDA approach. As shown in Fig. 13, we report the *mIoU* and *Dice* results under different (a) Center number *j*, (b) Number of neighbors *k*, and (c) Adjustment rate *φ*. In detail, *j* and *φ* are designed to determine how many clustering centers should be chosen for graph construction. Too many centers may lead to the graph overly fragmented, leading to unnecessary complexity, and too few centers may cause the graph too sparse, leading to possible missing of important information. Hence, suitable values of *j* and *φ* should be chosen to balance these two aspects. *k* is used to determine the number of nodes linked to each center. If *k* is too small, the information may be insufficient; if it is too large, it might introduce redundant information. The results shown in Fig. 13 (a), (b) and (c) demonstrate that our settings are the most optimal, achieving the best performance.

## 6 Potential future work: Exploring the possibility of Zero-shot learning from Subtomogram to Tomogram

In this section, we explore the feasibility of extending zero-shot learning from cryo-ET subtomograms to tomograms. Different from cryo-ET subutomograms each image in a tomogram contains multiple particles of different types, making it highly likely that molecular categories not being encountered during subtomogram training will appear. To address this problem, we define a new task, termed zero-shot cryo-ET segmentation, which aims to segment all the molecules with their categories using a model trained only with subtomograms. Our investigation is a preliminary exploration, but we hope it offers valuable insights for future research.

### Method details.

Given an input cryo-ET subtomogram *x_i_*, we set $y_{i}^{1}$ as its ground-truth segmentation mask, where 1 denotes the subtomogram contains only one type of molecule. To enable the model to detect unseen classes during the inference stage, similar to CLIP (Radford et al., 2021) in vision tasks, we have also built a similar vision-protein alignment model. As illustrated in Fig. 14 (a), we first feed the input subtomogram into the IBF module proposed in Sec. 3.2 and then pass the output to a segmentation model (Li et al., 2025b) based on a 3D Vision Transformer to extract visual features. This 3D Vision Transformer is an extension of the 2D Vision Transformer architecture, incorporating a novel 3D atrous convolution layer as the segmentor. For the protein feature embedding, we set the pre-trained protein language model (PLM) as the encoder. Given each input subtomogram, we also take the corresponding protein sequence as input and feed it into the encoder of the PLM. During training, we keep the PLM parameters frozen and use KL divergence (Kullback & Leibler, 1951) to quantify the difference between the visual and protein feature embeddings as the loss, optimizing only the segmentation model. For inference phase, the input cryo-ET tomogram is directly feed into IBF and the 3D segmentation model, and the output feature is further sent to the decoder of PLM to obtain the final prediction result $y_{\text{pred}}^{n}$, where *n* denotes the class numbers contained in the input tomogram.

### Experiments.

To test the proposed framework, we set the ESM-2 (Lin et al., 2022) pre-trained on ProtDescribe (Xu et al., 2023b) as the PLM, and use the SHREC2021 as the cryo-ET tomogram dataset for inference. Following the same simulation process in Sec. 5.1.2, we simulate 85 macromolecule complexes as the training datasets. Noted that there is no overlap in categories between the simulated dataset and SHREC2021. The whole experiment is conducted on 8 NVIDIA Tesla V100 Tensor Core GPU with a 32GB memory per card. The model is trained for 100 epochs with batch size of 1,024. The results are summarized in Table 15, and qualitative visualizations are provided in Fig. 16. The “Upperbond” setting refers to a fully supervised segmentation model that utilizes only the IBF module and the 3D Vision Transformer. Although our method achieves some promising results, the performance is still not optimal, and this research direction still requires further exploration.

## 7 Conclusion

In this paper, we propose a voxel-level unsupervised domain adaptation approach, termed Vox-UDA, for the cryo-ET subtomogram segmentation task. In detail, our Vox-UDA consists of a Noise Generation Module (NGM) and a denoised pseudo-labeling (DPL) strategy. NGM generates target-like Gaussian noise for the source domain data, while DPL uses an improved bilateral filter (IBF) to provide denoised target domain data for pseudo-labeling to boost the segmentation performance. Additionally, we propose Vox-ZSDA, a voxel-wise zero-shot domain adaptation approach to handle a more challenging scenario where target datasets are not available during training. Vox-ZSDA incorporates self-supervised graph learning strategy and dynamic graph contrastive learning technique to enhance the model’s ability on molecular structure segmentation. We have conducted large-scale experiments to demonstrate the prominent performance of our proposed methods and provide the first benchmark for both UDA and ZSDA of cryo-ET subtomogram segmentation tasks. Furthermore, we explore the possibility of zero-shot cryo-ET segmentation by transferring subtomogram to tomogram and propose a basic model structure and simple comparative experiments, hoping that these can inspire future research to explore unseen molecular detection.

## Figures and Tables

**Fig. 1:**
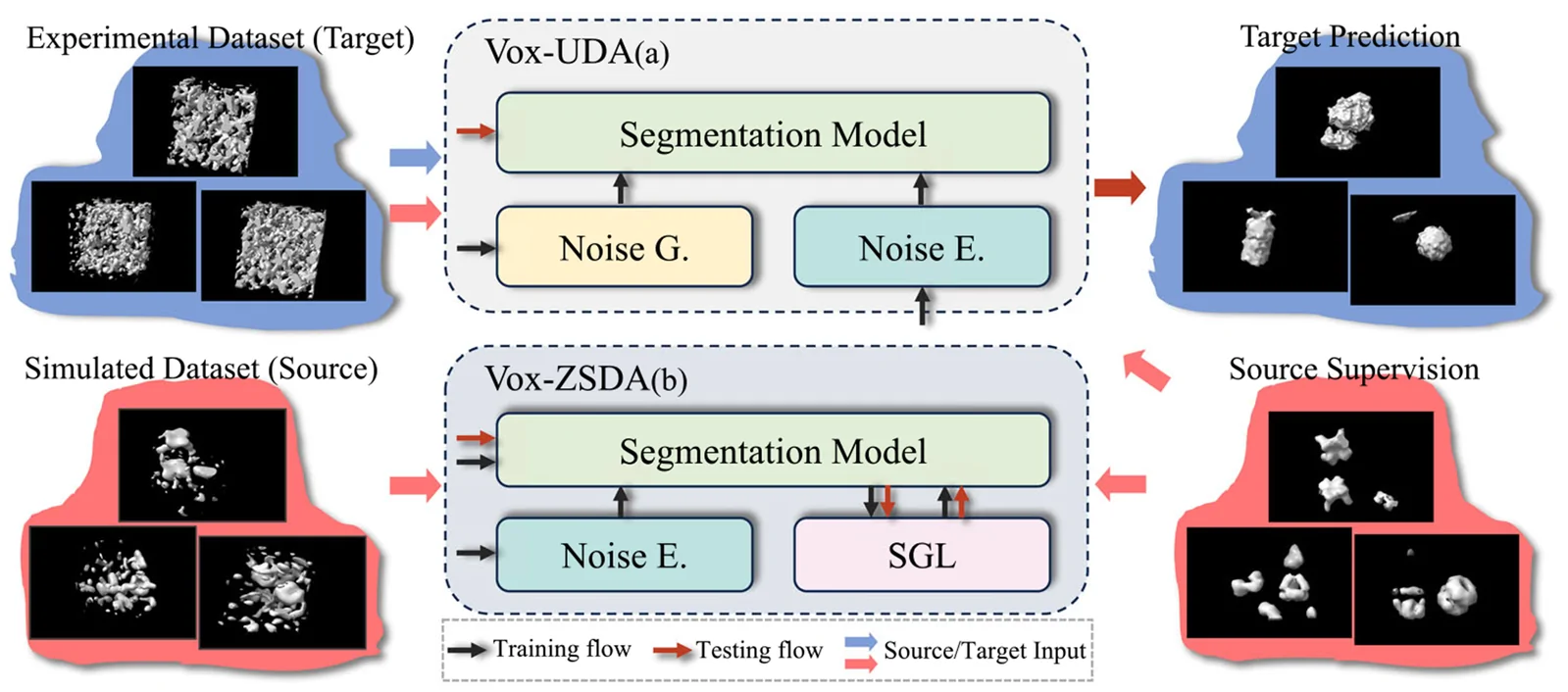
**Illustration of Vox-UDA (a) and Vox-ZSDA (b):** (a) Vox-UDA considers the unsupervised setting where target data can be utilized as input, the goal is to address noise differences between different domains. (b) Vox-ZSDA considers a more realistic task is considered, where target data is unavailable during training, aligning more closely with real-world scenarios. Best view in color

**Fig. 2:**
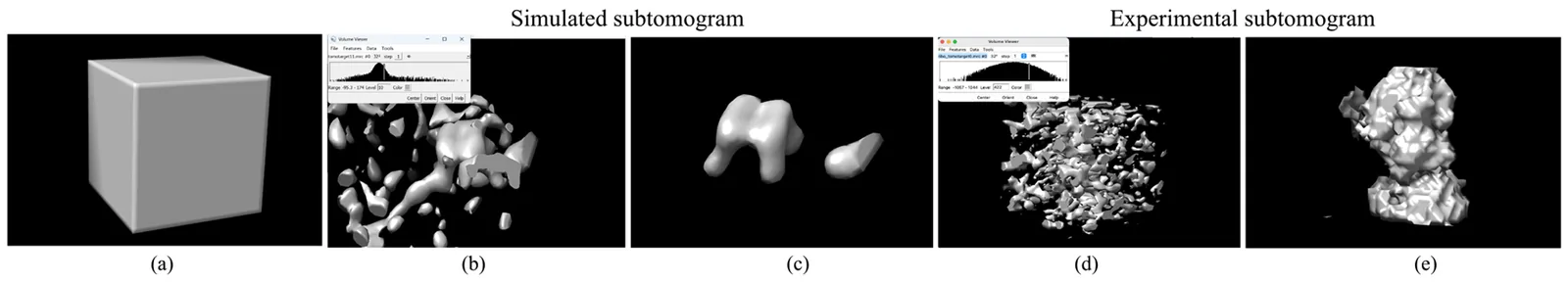
Examples of the subtomograms and their corresponding segmentation masks. (a) The voxel-space input representation used for both simulated and experimental subtomograms. To illustrate the differences in noise characteristics between the two domains, a fixed-range filter was applied separately to each type of data. (b–c) Simulated subtomogram after fixed-range noise filtering and its corresponding ground-truth segmentation mask. (d–e) Experimental subtomogram after fixed-range noise filtering and its corresponding ground-truth segmentation mask

**Fig. 3:**
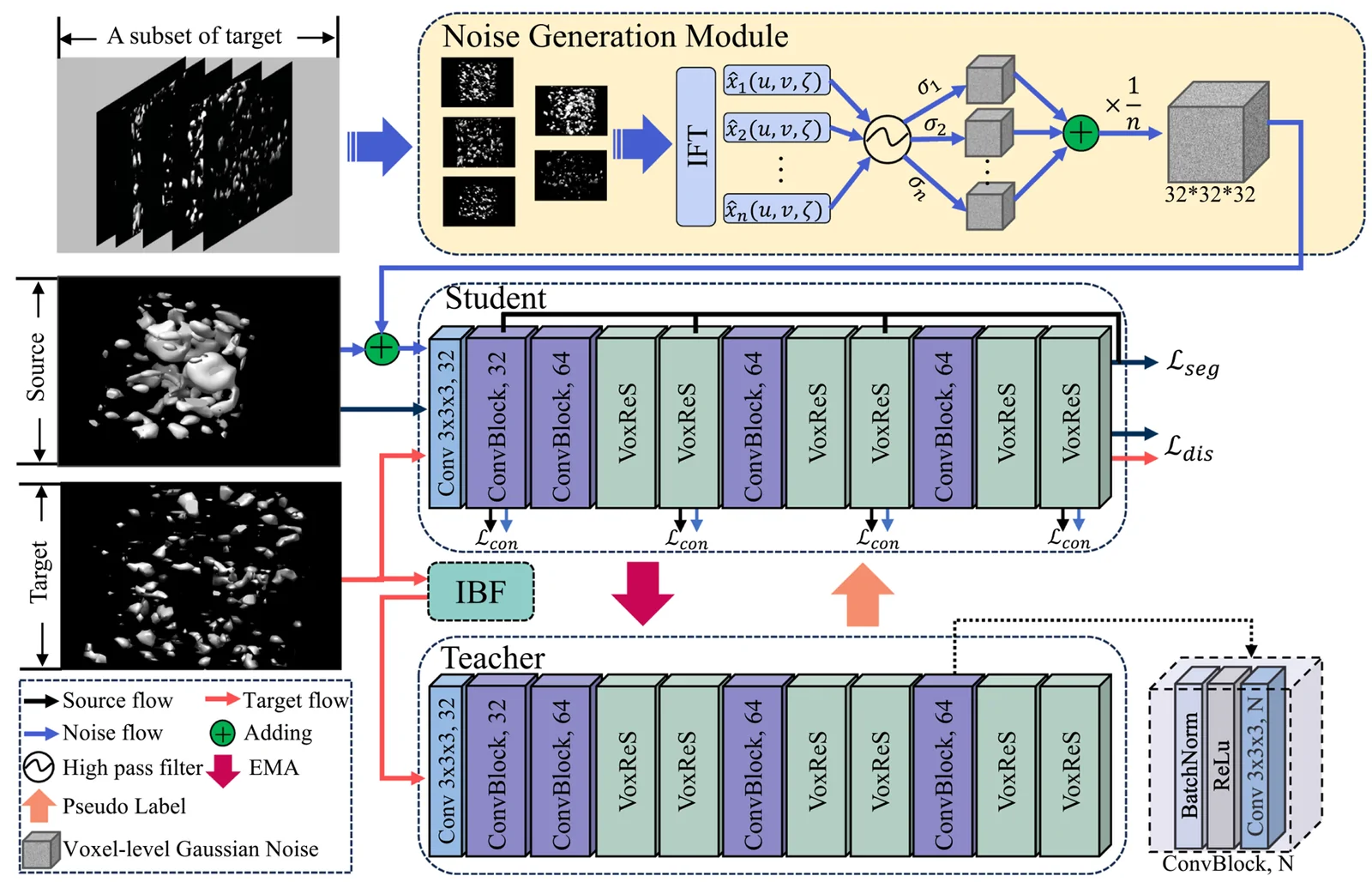
Overview of our proposed Vox-UDA framework. IBF denotes the improved bilateral filter. We use different colors to represent different flows. Best viewed in color

**Fig. 4:**
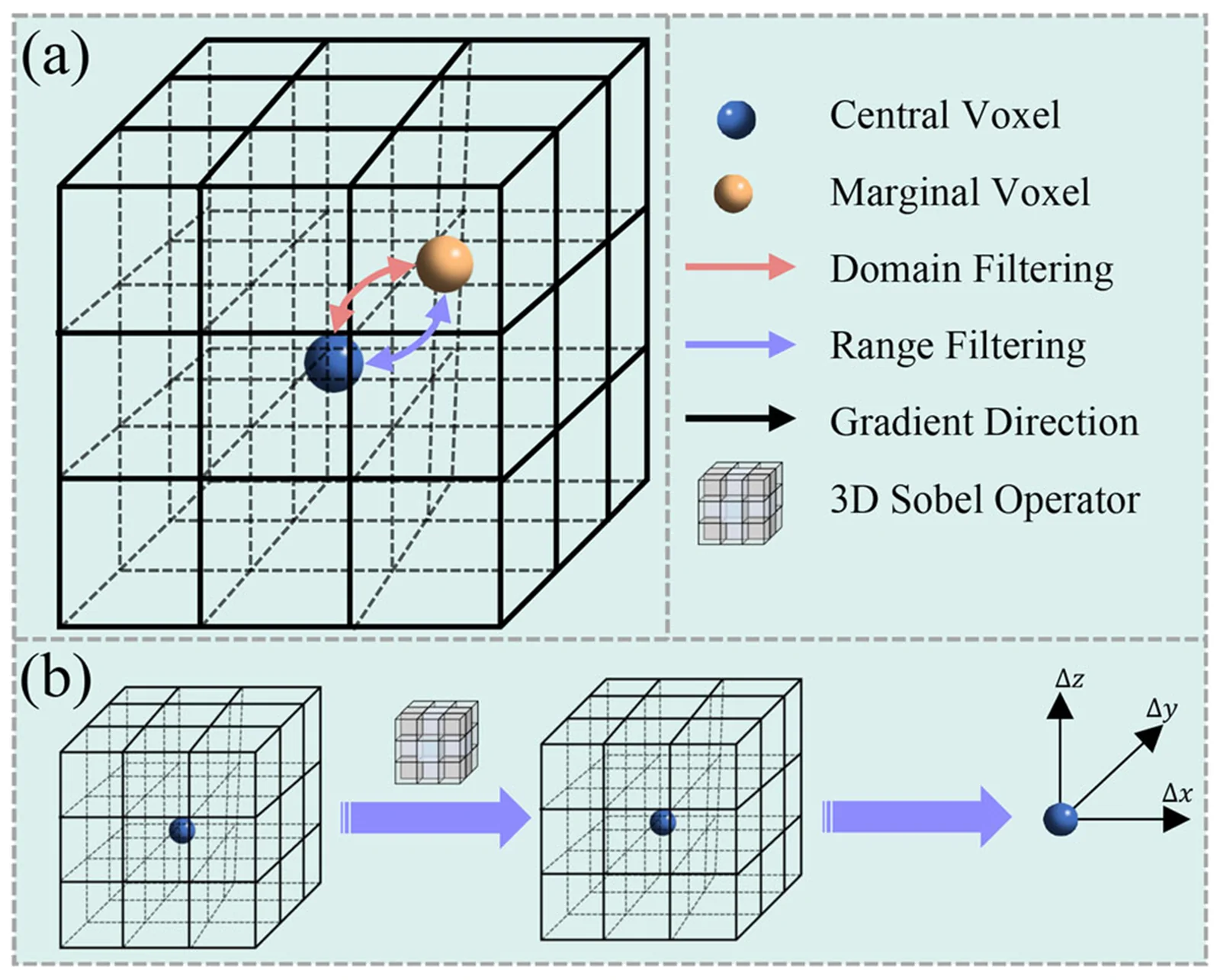
Proposed improved bilateral filter. (a) Both domain filtering and range filtering are applied to a sub-figure extracted from the input target subtomogram with size 3 × 3 × 3. (b) Computing 3D voxel gradients using the 3D Sobel operator to obtain gradient-based differences for range filtering

**Fig. 5:**
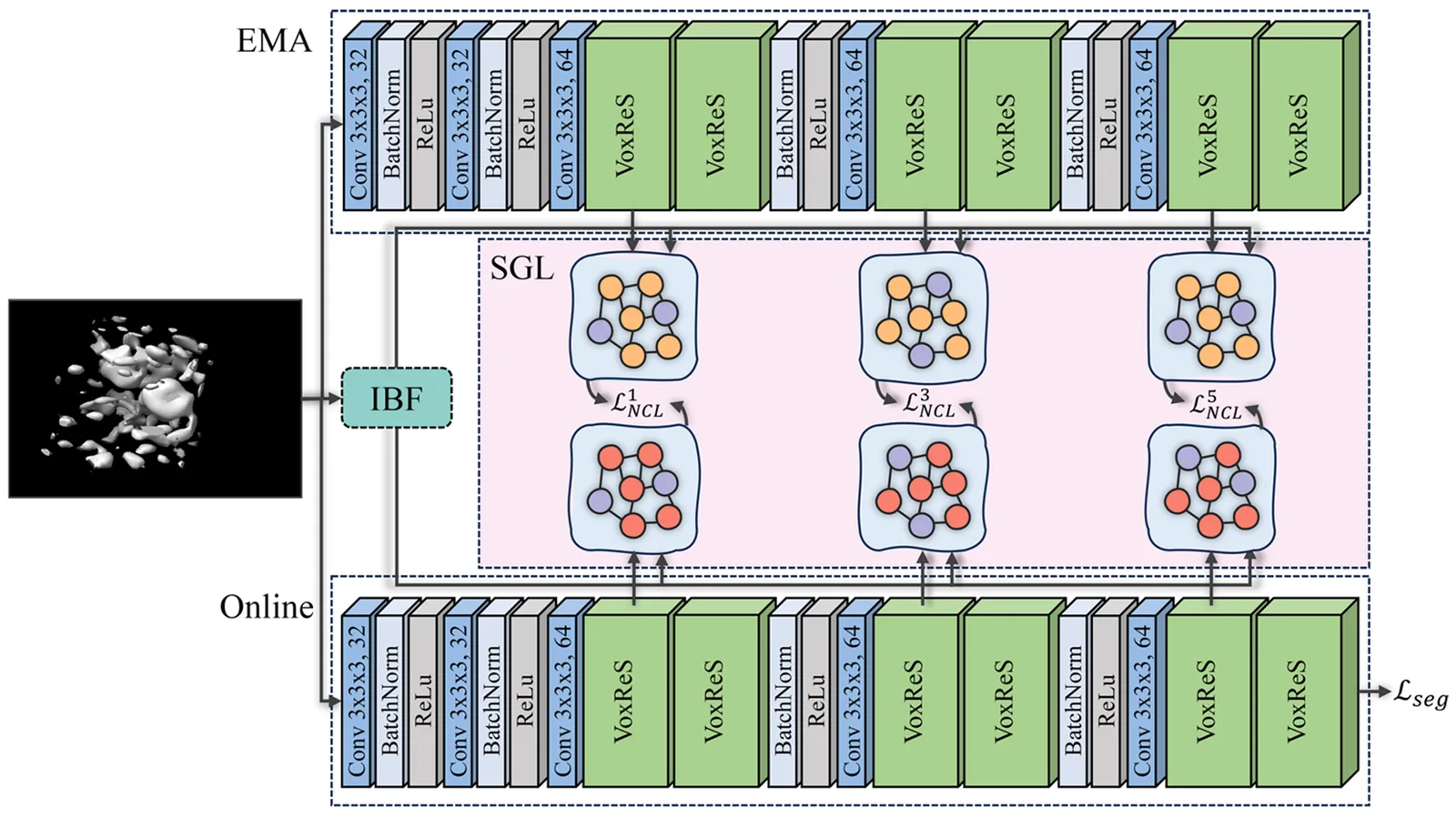
Illustration of the proposed Vox-ZSDA framework with self-supervised graph learning. In the SGL module, we use purple circles to represent the graph nodes participating in contrastive learning. Best viewed in color

**Fig. 6:**
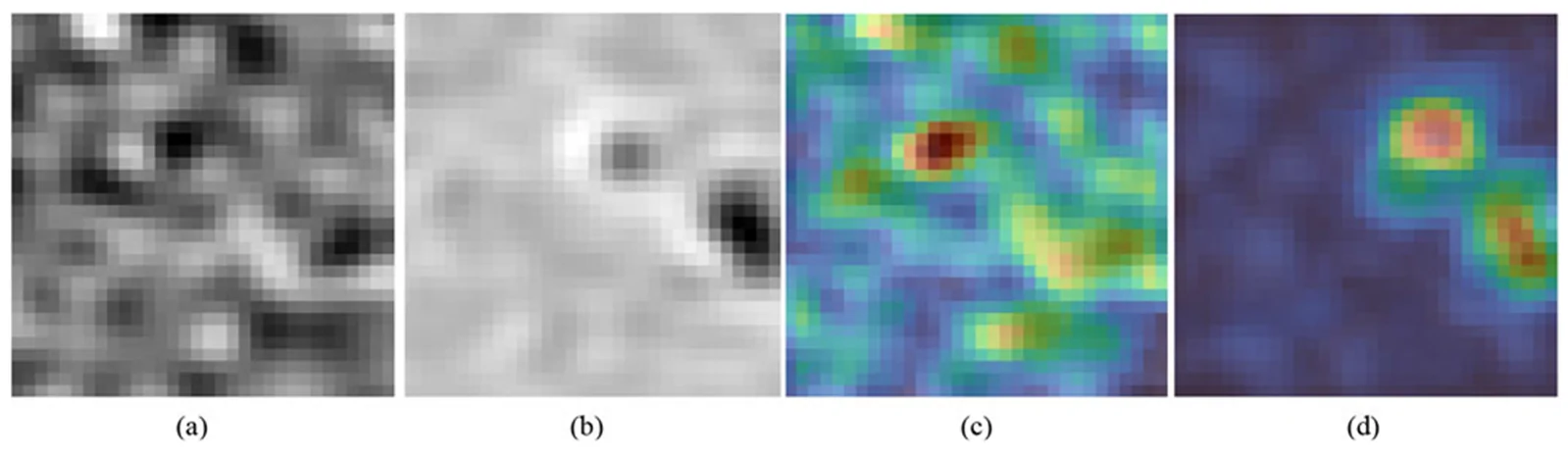
Visualization of the feature map. Since 3D volumetric data are difficult to visualize directly, we present representative z-slices for illustration. (a) Representative z-slice of the input cryo-ET subtomogram. (b) Particle regions. (c) Noise-cluster activation map. (d) Edge/texture-cluster activation map. The noise map mainly responds to high-frequency background regions, while the edge/texture map emphasizes particle boundaries and structural details

**Fig. 7:**
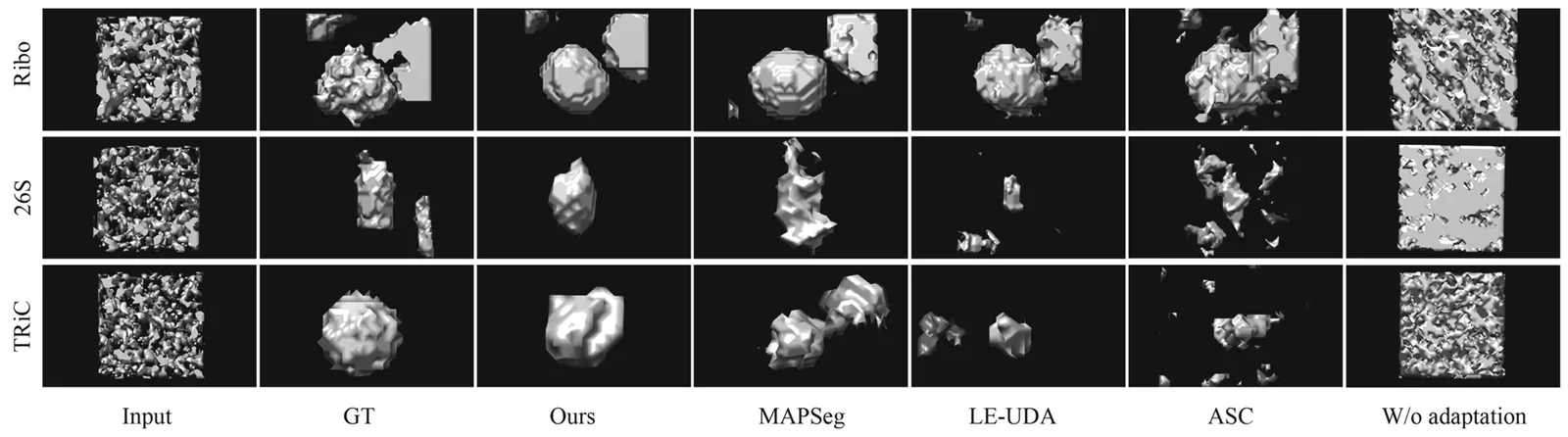
**Visualization of UDA subtomogram segmentation results** on Poly-GA dataset using 1bxn, 1f1b, and 1yg6 as the source datasets. We use UCSF Chimera (Pettersen et al., 2004) for 3D cryo-ET visualization

**Fig. 8:**
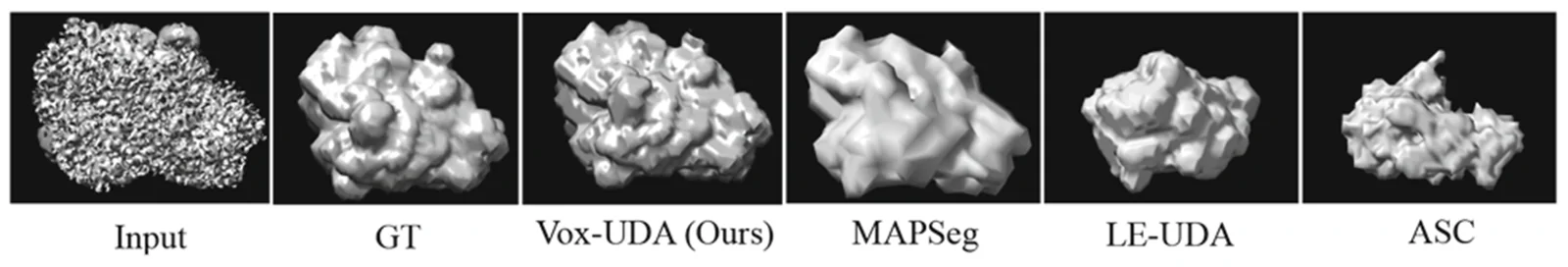
**Visualization of UDA subtomogram segmentation results** on *Mycoplasma pneumoniae* dataset using 1BXN, 1F1B, and 1YG6 as the source datasets.

**Fig. 9:**
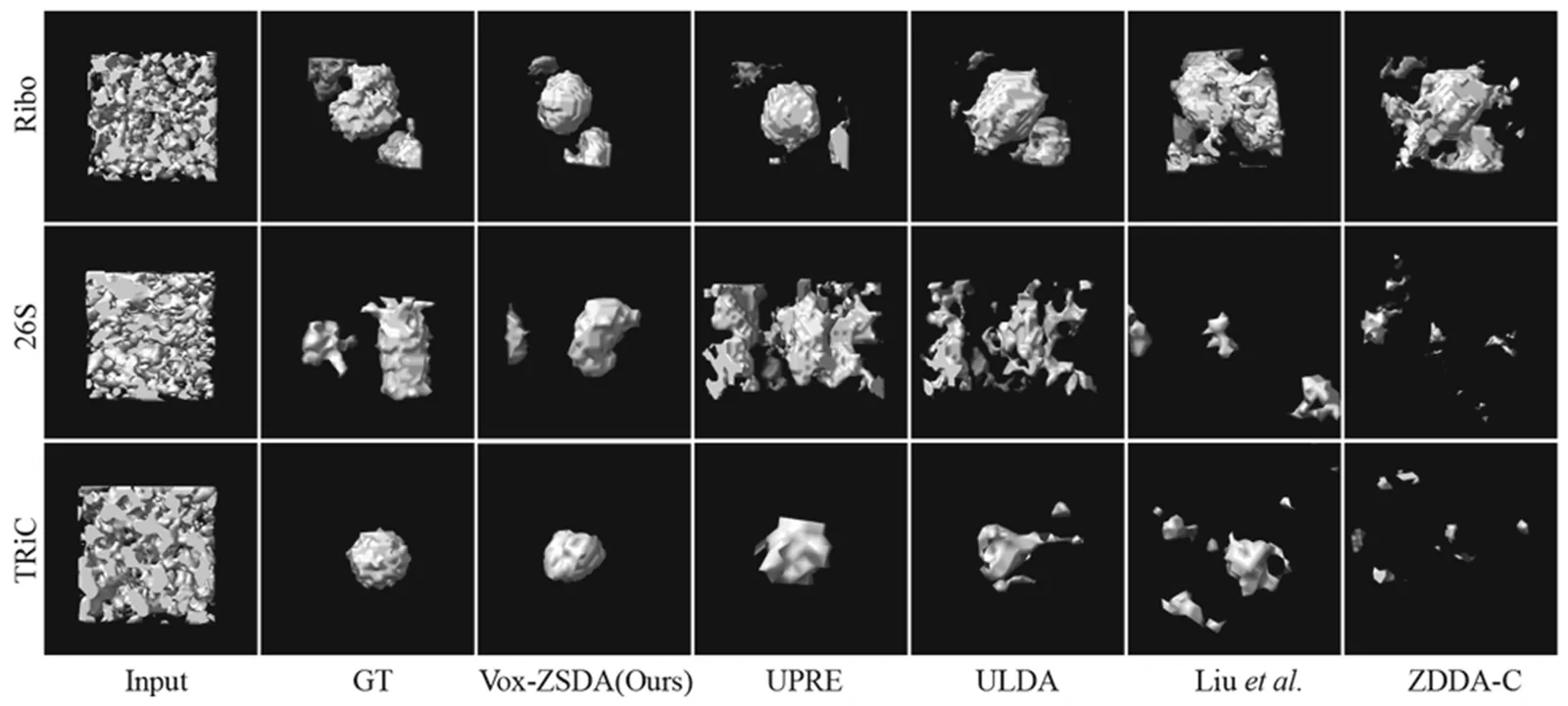
**Visualization of ZSDA subtomogram segmentation results** on Poly-GA dataset using 1bxn, 1f1b, and 1yg6 as the source datasets

**Fig. 10:**
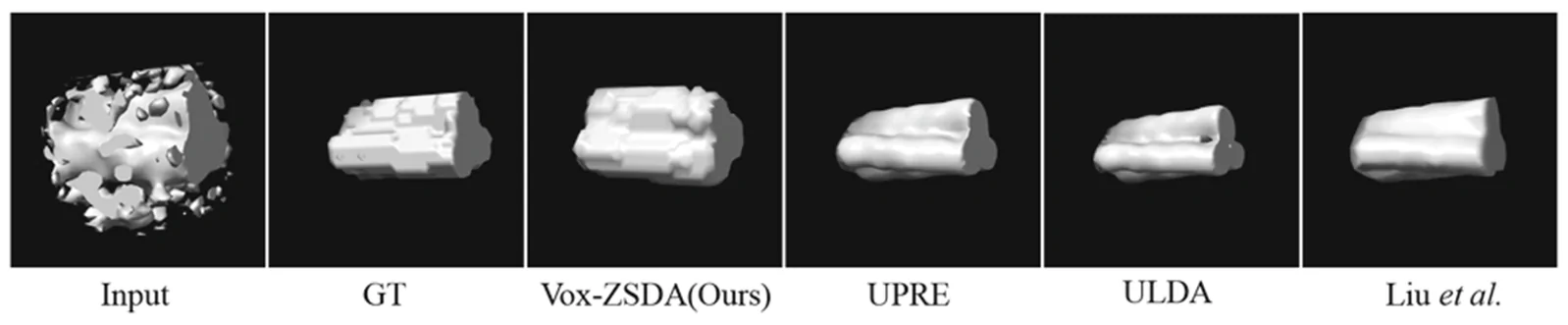
**Visualization of ZSDA subtomogram segmentation results** on ERWINIA dataset using 2BYU, 2H12 and 21DB as the source datasets

**Fig. 11:**
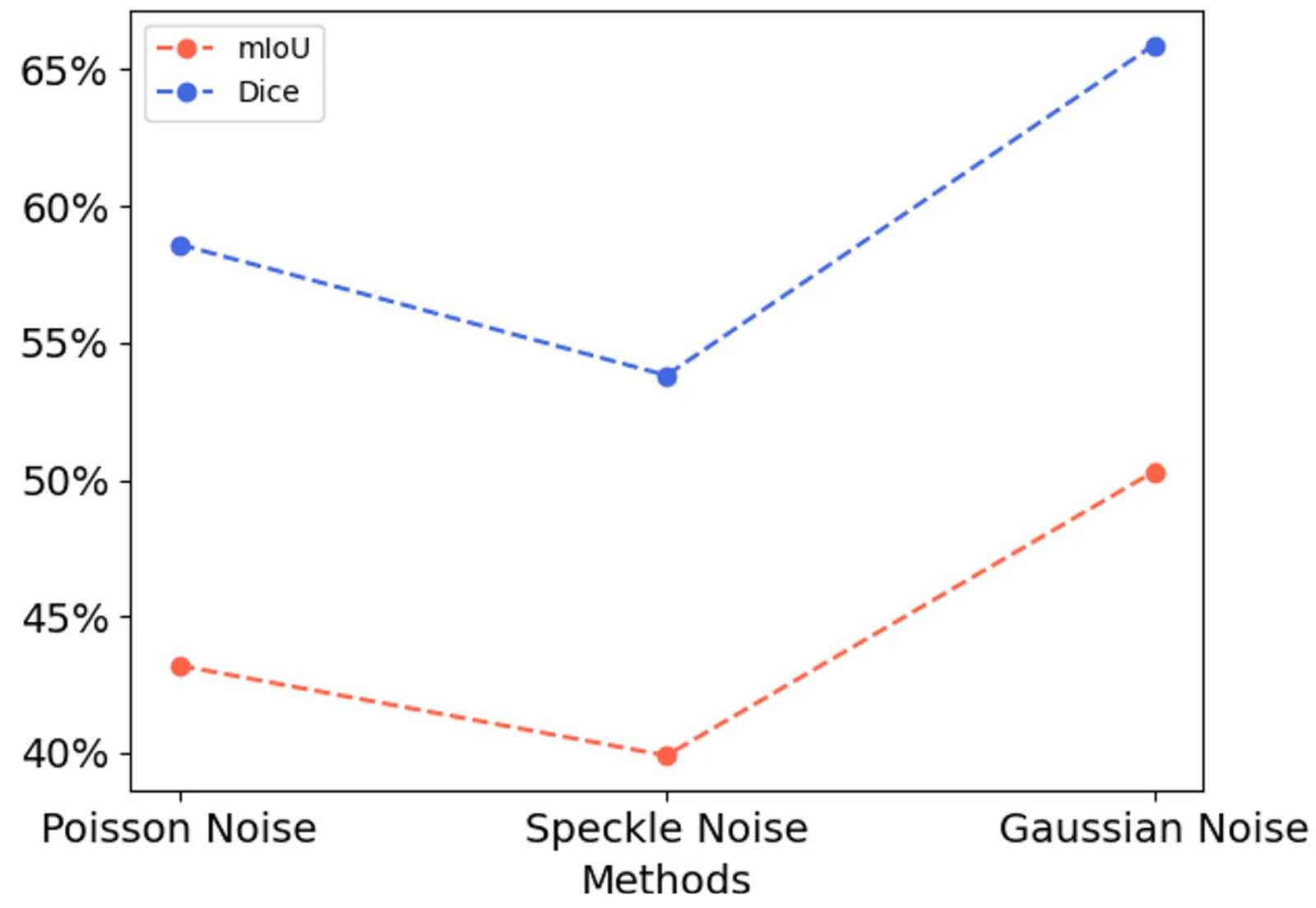
Ablation study of the proposed noise generation process. We set two different types of noise, Poisson noise and Speckle noise, to replace the Gaussian noise used in NGM for noise generation

**Fig. 12:**
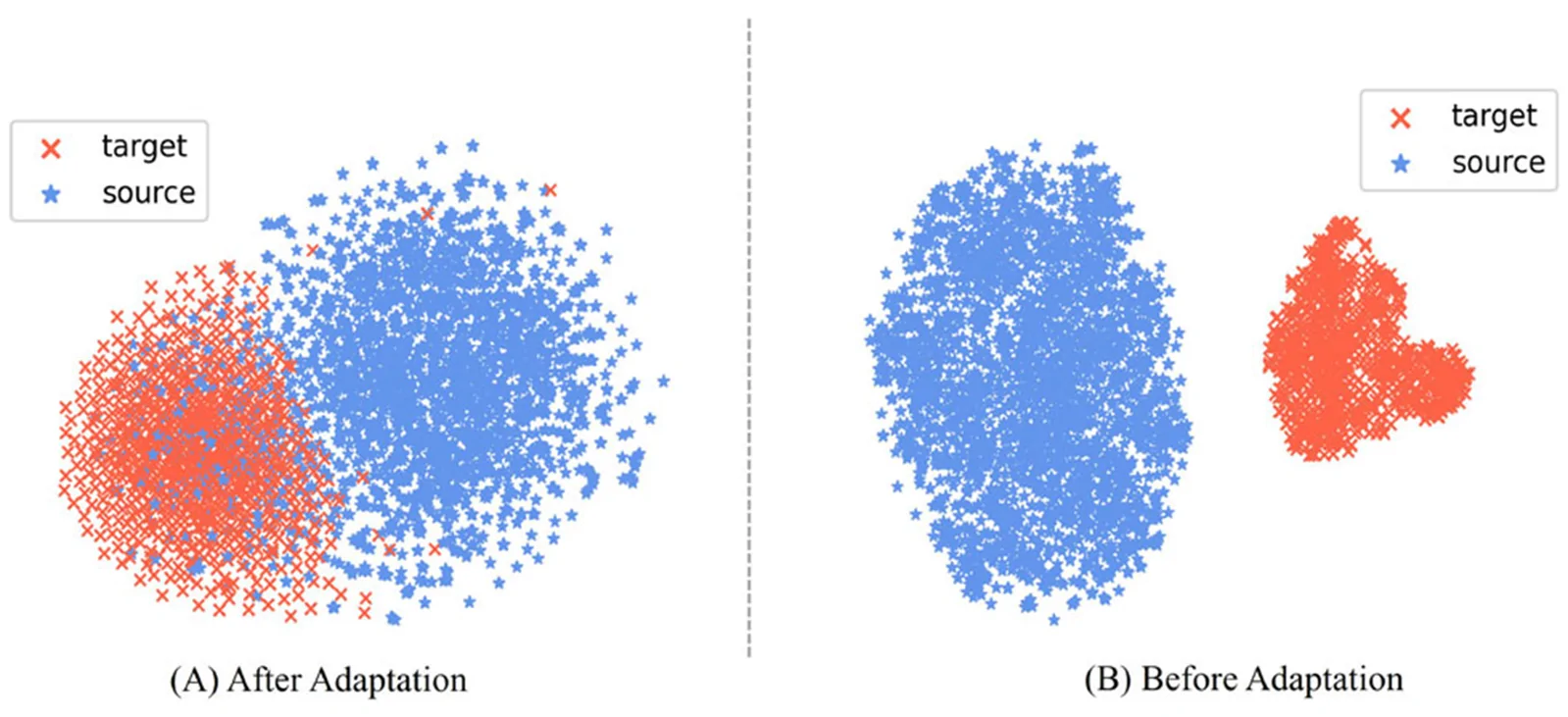
The t-SNE visualization of learned features. (a) After UDA. (b) Before UDA

**Fig. 13:**
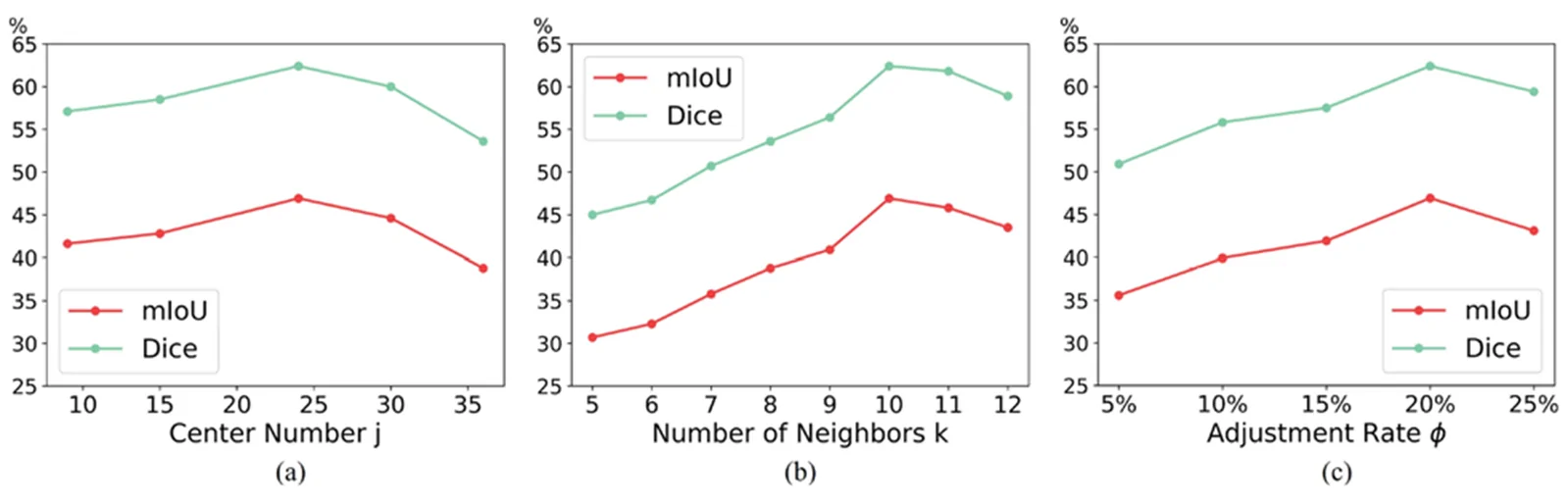
Ablation study on the hyperparameters of our proposed Vox-ZSDA on Poly-GA dataset

**Fig. 14:**
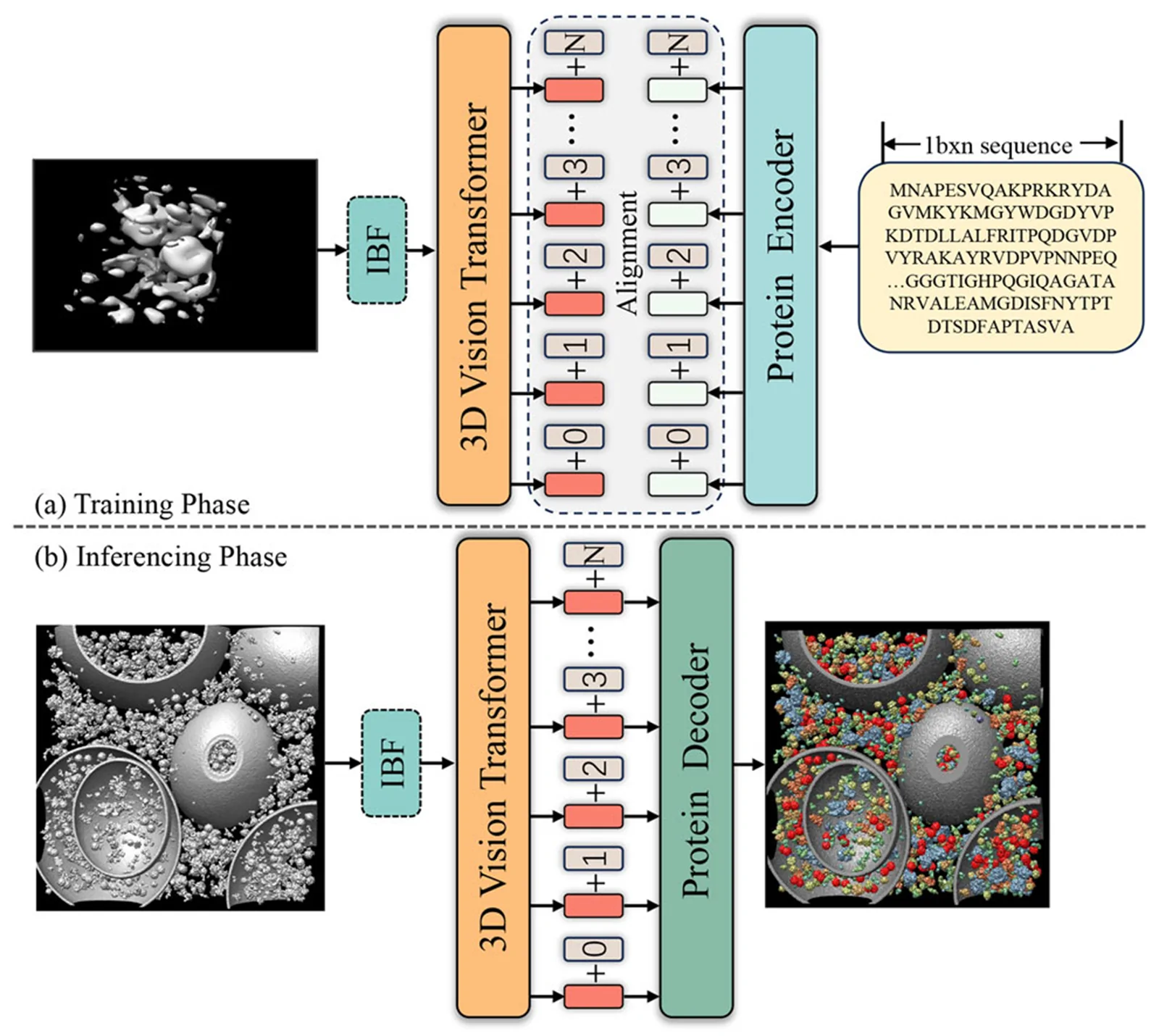
Simplified model design for zero-shot learning to transfer the subtomogram network to the tomogram segmentation task

**Fig. 15:**
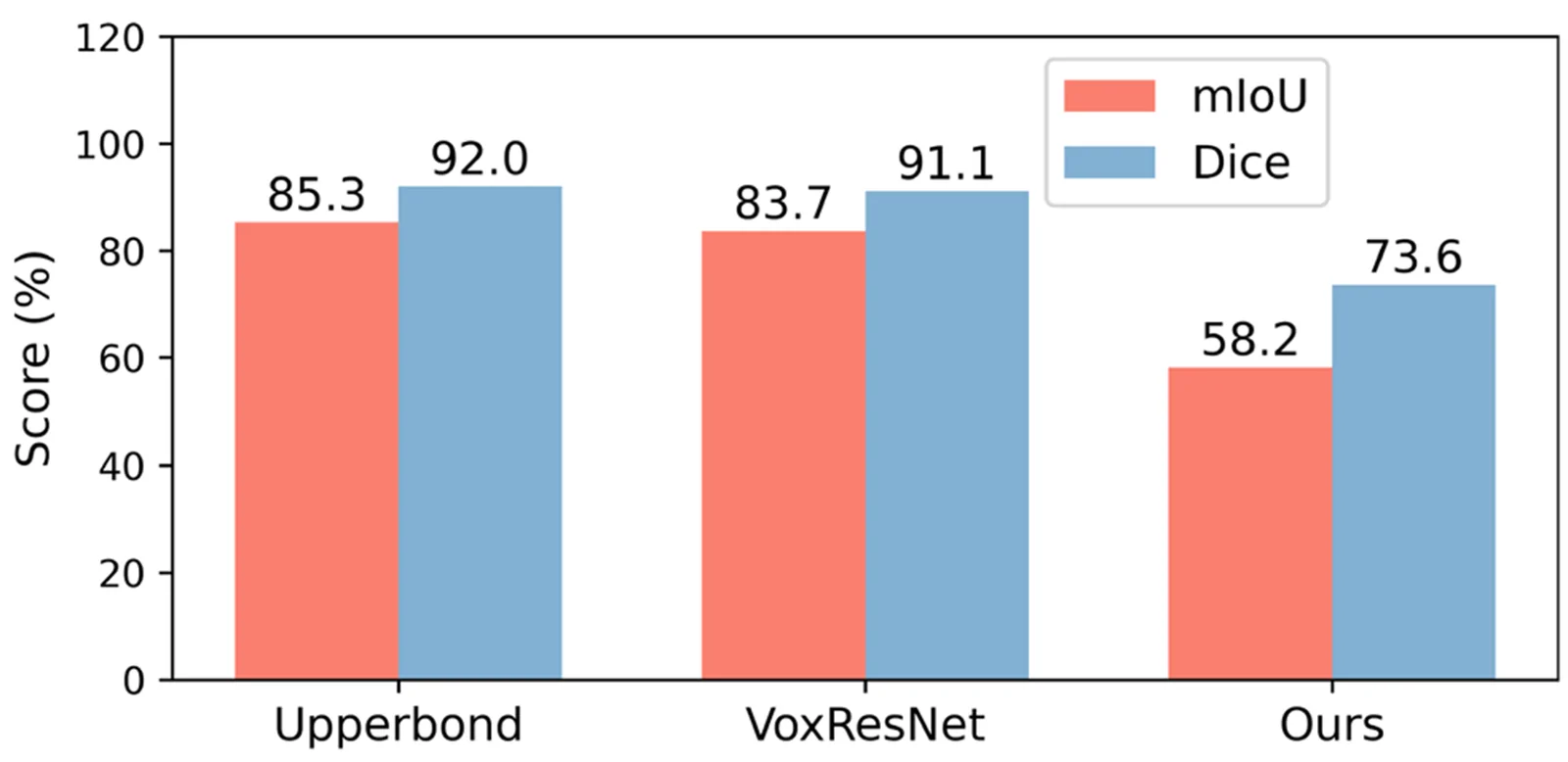
Experimental results on Zero-shot cryo-ET segmentation task

**Fig. 16:**
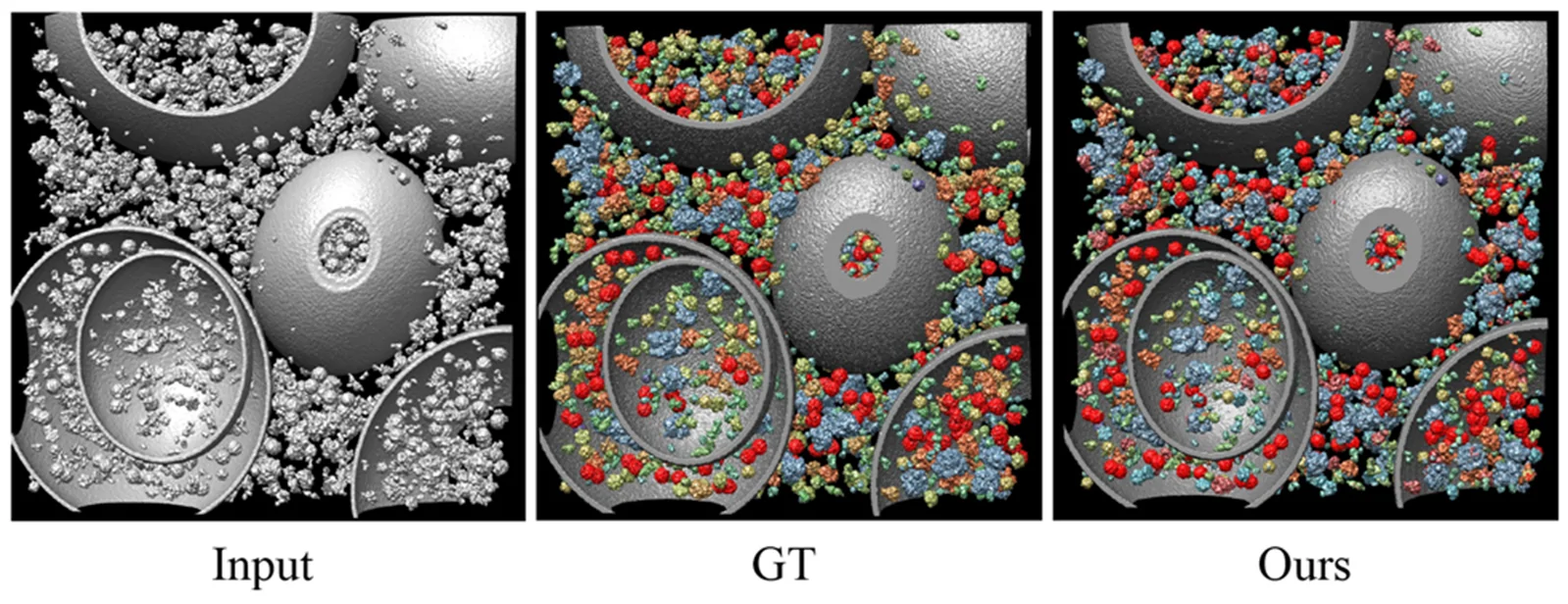
Visualization of Zero-shot cryo-ET tomogram segmentation results on SHREC2021 dataset

**Table 1:** Comparison of experimental results on UDA cryo-ET subtomogram segmentation between Vox-UDA and baselines using **Poly-GA** as target dataset. Best results are in bold font

Method	Source macromolecules: 1bxn, 1f1b, and 1yg6							
	mIoU	mIoU_*ribo*_	mIoU_26*S*_	mIoU_*T RiC*_	Dice	Dice*_ribo_*	Dice_26*S*_	Dice_*T RiC*_
w/o adaptation	9.7±_0.6_	11.1±_1.4_	2.6±_0.3_	2.6±_0.5_	17.4±_0.7_	19.9±_0.8_	5.1±_0.9_	5.0±_0.6_
Fully Supervised	55.8±_1.1_	57.9±_1.2_	36.7±_0.2_	48.8±_0.3_	71.0±_0.4_	73.1±_0.5_	53.0±_2.1_	63.1±_0.4_
DANN _[JMLR16]_ (Ganin et al., 2016)	38.4±_0.6_	43.0±_1.2_	6.5±_0.8_	11.7±_0.2_	53.0±_0.9_	59.1±_0.4_	11.2±_1.3_	16.8±_1.2_
PDAM _[CVPR20]_ (Liu et al., 2020)	39.8±_0.2_	43.3±_0.4_	13.8±_1.0_	22.6±_0.7_	55.1±_0.4_	59.6±_0.6_	21.5±_1.1_	31.9±_0.5_
Cryo-Shift _[Bioinfo.22]_ (Bandyopadhyay et al., 2022)	40.5±_0.2_	42.8±_0.3_	17.2±_0.1_	25.6±_0.5_	55.9±_0.4_	59.0±_0.2_	26.5±_0.6_	32.5±_0.3_
DAR-Unet _[JBHI22]_ (Yao et al., 2022)	41.0±_0.2_	44.3±_0.1_	19.0±_0.7_	25.6±_0.2_	57.1±_0.4_	58.1±_0.5_	29.9±_0.3_	37.2±_0.8_
LE-UDA _[TMI22]_ (Zhao et al., 2022)	41.5±_0.4_	44.7±_0.6_	18.4±_0.7_	23.9±_1.0_	56.8±_0.5_	61.0±_0.6_	28.4±_0.7_	32.6±_0.4_
ASC _[MICCAI23]_ (Xu et al., 2023a)	40.4±_0.5_	43.4±_0.9_	19.2±_0.4_	23.3±_0.5_	55.8±_0.3_	59.7±_0.4_	28.7±_0.8_	32.7±_0.3_
Zhao *et al.*_[WACV2024]_ (Zhao et al., 2024b)	41.8±_0.5_	43.9±_0.4_	19.9±_0.9_	27.8±_0.2_	57.4±_0.2_	60.2±_0.3_	30.7±_0.5_	30.3±_0.3_
DDSP _[MIA24]_ (Zheng et al., 2024c)	42.0±_0.7_	44.2±_0.2_	23.9±_0.4_	20.1±_0.7_	58.2±_0.2_	60.7±_0.3_	29.1±_0.4_	31.5±_0.3_
MAPSeg _[CVPR24]_ (Zhang et al., 2024a)	44.6±_0.3_	46.4±_0.4_	19.7±_0.1_	25.1±_0.2_	60.0±_0.6_	64.1±_1.3_	30.1±_0.7_	33.9±_0.1_
**Vox-UDA(_w_ NGM)**	48.5±_0.2_	50.6±_0.6_	**32.2**±_0.1_	38.6±_0.5_	64.4±_0.1_	66.8±_0.2_	**47.1**±_0.1_	51.5±_0.2_
**Vox-UDA(_w_ BF)**	49.1±_0.1_	50.4±_0.4_	30.5±_0.5_	39.2±_0.8_	64.5±_0.6_	67.1±_0.8_	46.9±_0.5_	50.7±_0.6_
**Vox-UDA(_w_ IBF)**	**50.3**±_0.1_	**53.8**±_0.3_	28.8±_0.7_	**41.3**±_0.3_	**65.9**±_0.2_	**68.5**±_0.6_	44.0±_1.0_	**52.8**±_0.2_

**Table 2:** Experimental results on UDA cryo-ET subtomogram segmentation under different dataset settings. Different from the results reported in Table 1, we set 2byu, 2h12, and 21db as the source datasets while using Poly-GA as target dataset

Method	Source macromolecules: 2byu, 2h12, and 21db							
	mIoU	mIoU_*ribo*_	mIoU_26*S*_	mIoU_*T RiC*_	Dice	Dice_*ribo*_	Dice_26*S*_	Dice_*T RiC*_
w/o adaptation	12.7±_0.2_	14.2±_0.5_	3.7±_0.9_	3.0±_0.4_	22.2±_0.5_	24.7±_0.3_	7.2±_0.3_	5.8±_0.3_
Fully Supervised	55.8±_1.1_	57.9±_1.2_	36.7±_0.2_	48.8±_0.3_	71.0±_0.4_	73.1±_0.5_	53.0±_2.1_	63.1±_0.4_
DANN _[JMLR16]_ (Ganin et al., 2016)	31.9±_0.8_	36.1±_0.7_	3.8±_1.3_	6.2±_1.2_	45.9±_0.5_	51.9±_0.3_	6.4±_0.4_	8.7±_0.2_
PDAM _[CVPR20]_ (Liu et al., 2020)	39.1±_0.5_	43.1±_0.3_	10.9±_0.6_	15.7±_0.7_	54.1±_0.2_	59.4±_0.2_	17.7±_0.2_	24.0±_0.2_
Cryo-Shift _[Bioinfo.22]_ (Bandyopadhyay et al., 2022)	39.5±_0.5_	41.9±_0.2_	23.2±_0.4_	26.1±_0.1_	55.6±_0.3_	58.5±_0.4_	35.6±_0.1_	28.8±_0.3_
DAR-Unet _[JBHI22]_ (Yao et al., 2022)	41.6±_0.5_	44.9±_0.3_	15.3±_0.1_	27.0±_0.3_	57.1±_0.3_	61.2±_0.2_	24.5±_0.7_	31.3±_0.1_
LE-UDA _[TMI22]_ (Zhao et al., 2022)	43.1±_1.4_	46.4±_0.6_	21.9±_0.3_	22.3±_1.4_	58.6±_1.3_	62.6±_0.7_	33.8±_1.3_	32.4±_0.9_
ASC _[MICCAI23]_ (Xu et al., 2023a)	41.7±_0.9_	45.2±_0.2_	24.7±_0.3_	13.3±_1.3_	56.9±_1.0_	61.2±_1.2_	38.1±_0.6_	19.8±_1.2_
Zhao *et al.*_[WACV2024]_ (Zhao et al., 2024b)	42.5±_0.1_	45.1±_0.3_	22.0±_0.1_	30.9±_0.2_	58.3±_0.5_	61.5±_0.4_	33.4±_0.1_	31.6±_0.3_
DDSP _[MIA24]_ (Zheng et al., 2024c)	43.1±_0.2_	46.6±_0.4_	19.1±_0.1_	23.4±_0.1_	58.7±_0.1_	63.0±_0.5_	30.4±_0.2_	35.2±_0.9_
MAPSeg _[CVPR24]_ (Zhang et al., 2024a)	44.2±_0.6_	47.7±_1.0_	22.6±_0.7_	23.2±_0.4_	59.9±_0.6_	63.9±_0.4_	34.5±_1.2_	33.4±_0.8_
**Vox-UDA(w NGM)**	47.5±_0.2_	50.1±_0.1_	27.5±_0.2_	34.7±_0.7_	63.2±_0.2_	66.3±_0.5_	41.0±_0.4_	46.8±_1.0_
**Vox-UDA(w BF)**	48.0±_0.1_	50.3±_0.4_	27.8±_0.6_	34.4±_0.6_	63.8±_0.1_	66.7±_0.2_	39.9±_0.3_	47.0±_0.7_
**Vox-UDA(w IBF)**	**49.5**±_0.3_	**52.4**±_0.3_	**28.3**±_0.4_	**35.1**±_0.6_	**65.2**±_0.2_	**68.9**±_0.2_	**41.3**±_0.4_	**47.7**±_0.1_

**Table 3:** Experimental results on UDA cryo-ET subtomogram segmentation using ***Mycoplasma pneumoniae*** and **Erwinia** as target datasets. We set Setting I: 1bxn, 1f1b, and 1yg6; and Setting II: 2byu, 2h12 and 21db as the source datasets, respectively

Method	*Mycoplasma pneumoniae*				Erwinia			
	Setting I		Setting II		Setting I		Setting II	
	mIoU	Dice	mIoU	Dice	mIoU	Dice	mIoU	Dice
w/o adaptation	37.8±_0.3_	54.8±_1.2_	38.1±_0.5_	55.2±_1.1_	28.3±_0.1_	44.2±_0.5_	23.7±_0.9_	38.3±_1.3_
Fully Supervised	99.1±_0.2_	99.5±_0.1_	99.1±_0.6_	99.5±_0.1_	95.4±_1.0_	97.6±_0.4_	95.4±_1.3_	97.6±_0.7_
DANN _[JMLR16]_ (Ganin et al., 2016)	45.4±_0.5_	62.5±_0.2_	62.3±_0.5_	76.8±_0.3_	37.6±_1.0_	54.7±_1.4_	35.3±_0.2_	52.2±_1.2_
PDAM _[CVPR20]_ (Liu et al., 2020)	82.0±_0.4_	90.1±_0.3_	78.2±_0.7_	87.8±_1.2_	60.9±_0.5_	75.7±_0.7_	58.6±_1.2_	73.9±_0.8_
Cryo-Shift _[Bioinfo.22]_ (Bandyopadhyay et al., 2022)	83.4±_0.2_	91.0±_0.1_	88.7±_0.4_	94.0±_0.6_	66.2±_0.3_	79.6±_0.3_	67.5±_0.1_	80.6±_1.1_
DAR-Unet _[JBHI22]_ (Yao et al., 2022)	89.7±_1.2_	94.6±_0.7_	91.6±_0.1_	95.9±_0.1_	76.6±_0.4_	86.8±_0.3_	75.6±_0.2_	86.1±_0.1_
LE-UDA _[TMI22]_ (Zhao et al., 2022)	92.9±_0.8_	96.3±_0.8_	93.0±_0.4_	96.4±_0.7_	79.9±_0.4_	88.8±_1.3_	77.6±_0.7_	87.4±_2.1_
ASC _[MICCAI23]_ (Xu et al., 2023a)	87.9±_0.5_	93.5±_1.2_	91.3±_0.7_	95.4±_1.0_	72.7±_1.3_	84.2±_0.4_	73.8±_0.8_	84.9±_1.0_
Zhao *et al.*_[WACV2024]_ (Zhao et al., 2024b)	92.0±_0.1_	95.9±_0.1_	92.9±_0.2_	96.3±_0.5_	78.0±_0.4_	87.6±_0.2_	77.2±_0.7_	87.1±_1.4_
DDSP _[MIA24]_ (Zheng et al., 2024c)	93.5±_0.4_	96.7±_0.2_	93.2±_0.2_	96.5±_0.1_	80.2±_0.3_	89.0±_0.6_	79.1±_0.4_	88.3±_0.4_
MAPSeg _[CVPR24]_ (Zhang et al., 2024a)	94.9±_0.4_	97.4±_0.6_	94.0±_0.3_	96.9±_0.5_	81.8±_1.5_	90.0±_0.8_	79.5±_0.6_	88.6±_0.1_
**Vox-UDA(w NGM)**	94.4±_0.2_	97.1±_0.5_	94.1±_0.1_	97.0±_0.3_	83.1±_0.4_	90.8±_0.8_	80.0±_1.2_	88.8±_0.3_
**Vox-UDA(w BF)**	95.0±_0.3_	97.5±_0.4_	95.1±_0.6_	97.5±_0.5_	85.3±_0.2_	92.0±_0.3_	82.0±_1.1_	90.1±_0.7_
**Vox-UDA(w IBF)**	**98.9**±_0.1_	**99.4**±_0.2_	**97.4**±_0.3_	**98.7**±_0.4_	**88.7**±_0.4_	**94.0**±_0.8_	**83.5**±_1.2_	**91.0**±_0.6_

**Table 4:** Comparison of experimental results on ZSDA cryo-ET subtomogram segmentation between Vox-ZSDA and baselines using **Poly-GA** as target dataset. Best results are in bold font

Method	Source macromolecules: 1bxn, 1f1b, and 1yg6							
	mIoU	mIoU_*ribo*_	mIoU_26*S*_	mIoU_*T RiC*_	Dice	Dice_*ribo*_	Dice_26*S*_	Dice_*T RiC*_
w/o adaptation	9.7±_0.6_	11.1±_1.4_	2.6±_0.3_	2.6±_0.5_	17.4±_0.7_	19.9±_0.8_	5.1±_0.9_	5.0±_0.6_
Fully Supervised	55.8±_1.1_	57.9±_1.2_	36.7±_0.2_	48.8±_0.3_	71.0±_0.4_	73.1±_0.5_	53.0±_2.1_	63.1±_0.4_
ZSDA _[ECCV20]_ (Wang & Jiang, 2020)	30.1±_0.4_	33.1±_0.3_	8.5±_0.5_	13.0±_1.0_	43.9±_0.7_	48.1±_0.5_	13.3±_0.1_	20.4±_0.4_
ZDDA-C _[TPAMI21]_ (Kutbi et al., 2021)	34.4±_0.2_	37.5±_0.5_	12.0±_0.1_	18.6±_1.2_	49.3±_1.0_	53.3±_0.8_	19.3±_1.0_	28.1±_1.4_
P∅DA _[ICCV23]_ (Fahes et al., 2023)	37.9±_0.3_	41.0±_0.5_	18.5±_0.3_	17.1±_0.7_	53.7±_1.4_	57.5±_0.6_	30.5±_0.8_	27.7±_0.4_
Liu *et al.*_[TMM24]_ (Liu et al., 2024)	37.7±_1.5_	40.0±_0.9_	23.5±_1.2_	22.3±_1.3_	54.0±_0.2_	56.8±_0.5_	**37.6**±_1.1_	34.8±_0.7_
ULDA _[CVPR24]_ (Yang et al., 2024)	41.4±_0.6_	44.4±_0.4_	18.8±_1.3_	26.1±_0.7_	57.3±_0.5_	61.0±_1.3_	29.7±_0.4_	38.1±_0.6_
UPRE _[ICCV25]_ (Zhang et al., 2025)	42.1±_0.3_	45.3±_0.2_	19.7±_0.8_	27.5±_0.3_	58.7±_0.1_	61.9±_0.5_	30.0±_1.2_	39.4±_0.3_
**Vox-ZSDA**	**46.9**±_0.5_	**50.2**±_0.2_	**22.1**±_0.3_	**30.1**±_0.7_	**62.4**±_0.6_	**66.3**±_0.4_	34.7±_0.7_	**41.6**±_0.1_

**Table 5:** Experimental results on ZSDA cryo-ET subtomogram segmentation under different dataset settings. Different from the results reported in Table 4, we set 2byu, 2h12, and 21db as the source datasets and using Poly-GA as target dataset

	Source macromolecules: 2byu, 2h12, and 21db							
Method	mIoU	mIoU_*ribo*_	mIoU_26*S*_	mIoU_*T RiC*_	Dice	Dice_*ribo*_	Dice_26*S*_	Dice_*T RiC*_
w/o adaptation	12.7±_0.2_	14.2±_0.5_	3.7±_0.9_	3.0±_0.4_	22.2±_0.5_	24.7±_0.3_	7.2±_0.3_	5.8±_0.3_
Fully Supervised	55.8±_1.1_	57.9±_1.2_	36.7±_0.2_	48.8±_0.3_	71.0±_0.4_	73.1±_0.5_	53.0±_2.1_	63.1±_0.4_
ZSDA _[ECCV20]_ (Wang & Jiang, 2020)	30.8±_0.5_	33.2±_1.2_	16.9±_1.3_	13.6±_0.1_	46.2±_0.3_	49.4±_1.1_	28.7±_0.2_	22.0±_1.1_
ZDDA-C _[TPAMI21]_ (Kutbi et al., 2021)	32.4±_1.5_	35.0±_0.5_	17.8±_0.9_	13.9±_1.3_	48.0±_1.2_	51.3±_0.5_	29.9±_0.5_	22.8±_0.5_
P∅DA _[ICCV23]_ (Fahes et al., 2023)	37.3±_0.2_	40.9±_1.0_	8.6±_1.5_	20.1±_0.7_	52.3±_0.9_	57.1±_1.2_	14.7±_1.4_	28.9±_0.6_
Liu *et al.*_[TMM24]_ (Liu et al., 2024)	36.1±_0.6_	38.8±_0.4_	20.2±_1.4_	18.2±_0.3_	52.2±_0.9_	55.4±_0.7_	33.2±_1.1_	29.6±_0.3_
ULDA _[CVPR24]_ (Yang et al., 2024)	40.5±_0.2_	44.1±_1.0_	18.7±_0.2_	15.7±_0.8_	56.2±_0.3_	60.6±_1.3_	30.5±_1.5_	25.5±_0.5_
UPRE _[ICCV25]_ (Zhang et al., 2025)	42.0±_0.5_	44.2±_0.1_	23.9±_1.5_	23.3±_0.1_	58.2±_0.4_	61.4±_1.0_	31.7±_0.4_	27.1±_0.2_
**Vox-ZSDA**	**45.1**±_0.6_	**48.2**±_0.7_	**25.7**±_1.2_	**25.9**±_0.4_	**60.7**±_0.3_	**64.3**±_0.5_	**38.3**±_0.7_	**37.7**±_1.0_

**Table 6:** Experimental results on ZSDA cryo-ET subtomogram segmentation using ***Mycoplasma pneumoniae*** and **Erwinia** as target dataset. We set Setting I: 1bxn, 1f1b, and 1yg6; and Setting II: 2byu, 2h12 and 21db as the source datasets, respectively

Method	*Mycoplasma pneumoniae*				Erwinia			
	Setting I		Setting II		Setting I		Setting II	
mIoU	Dice	mIoU	Dice	mIoU	Dice	mIoU	Dice
w/o adaptation	37.8±_0.3_	54.8±_1.2_	38.1±_0.5_	55.2±_1.1_	28.3±_0.1_	44.2±_0.5_	23.7±_0.9_	38.3±_1.3_
Fully Supervised	99.1±_0.2_	99.5±_0.1_	99.1±_0.6_	99.5±_0.1_	95.4±_1.0_	97.6±_0.4_	95.4±_1.3_	97.6±_0.7_
ZSDA _[ECCV20]_ (Wang & Jiang, 2020)	73.3±_0.6_	84.6±_1.0_	71.1±_0.7_	83.1±_0.2_	52.8±_1.4_	69.1±_0.4_	48.6±_1.1_	65.7±_0.4_
ZDDA-C _[TPAMI21]_ (Kutbi et al., 2021)	79.1±_1.2_	88.3±_1.0_	82.3±_1.2_	90.3±_0.3_	64.0±_1.1_	78.0±_0.2_	61.8±_0.2_	76.5±_0.1_
P∅DA _[ICCV23]_ (Fahes et al., 2023)	82.7±_0.9_	90.2±_1.3_	83.1±_1.5_	90.8±_0.4_	69.6±_1.0_	82.2±_1.1_	64.8±_0.5_	78.7±_0.3_
Liu *et al.*_[TMM24]_ (Liu et al., 2024)	85.1±_0.3_	92.0±_1.5_	86.5±_0.1_	92.8±_0.3_	72.6±_0.3_	84.1±_1.3_	70.1±_0.8_	82.4±_0.8_
ULDA _[CVPR24]_ (Yang et al., 2024)	75.6±_1.4_	86.3±_0.8_	73.4±_0.7_	84.6±_1.0_	75.6±_1.4_	86.3±_0.8_	73.4±_0.7_	84.6±_1.0_
UPRE _[ICCV25]_ (Zhang et al., 2025)	87.0±_0.2_	93.1±_0.4_	87.8±_0.2_	93.5±_0.1_	78.7±_0.1_	88.1±_0.5_	75.9±_0.4_	86.3±_1.3_
**Vox-ZSDA**	**93.3**±_1.1_	**96.5**±_0.4_	**92.5**±_0.7_	**96.1**±_0.3_	**81.8**±_1.3_	**90.0**±_0.8_	**78.1**±_1.2_	**87.7**±_0.7_

**Table 7:** Ablation study on the IBF module on Poly-GA dataset using 1bxn, 1f1b, and 1yg6 as source dataset

Method	mIoU	Dice
Vox-UDA(BF)	49.1	64.5
Vox-UDA(ABF)	49.5	65.0
**Vox-UDA(IBF)**	**50.3**	**65.9**

**Table 8:** Ablation study on the components of our proposed Vox-UDA on Poly-GA dataset using 1bxn, 1f1b, and 1yg6 as source dataset. Best results are in bold font

Method	mIoU	Dice
Baseline	31.9	45.9
w/o NGM	41.9	57.5
w/o PL	46.2	61.8
**Vox-UDA(Ours)**	**50.3**	**65.9**

**Table 9:** Ablation study on the hyperparameters of our proposed Vox-UDA on Poly-GA dataset. $N_{\text{sample}},\rho$ and $\lambda_{n}$ denote the sampled number of target data used for noise generation, the high-pass filter rate and the weights for different losses, respectively

Source macromolecules: 1bxn, 1f1b, and 1yg6														
N_*sample*_	mIoU	Dice	*ρ*	mIoU	Dice	*λ*_1_ → *λ*_4_	mIoU	Dice	*σ_d_*	mIoU	Dice	*σ_r_*	mIoU	Dice
5	41.2±_0.2_	58.4±_0.1_	8.4%	41.7±_0.3_	57.3±_0.1_	[0.1, 0.1, 0.4, 0.4]	44.4±_0.0_	60.2±_0.0_	100	49.3±_0.1_	64.9±_0.1_	0.8	47.5±_0.1_	63.2±_0.0_
**10**	**50.3**±_0.1_	**65.9**±_0.2_	17.8%	43.5±_0.3_	59.6±_0.2_	**[0.2, 0.2, 0.3, 0.3]**	**50.3**±_0.1_	**65.9**±_0.2_	**120**	**50.3**±_0.1_	**65.9**±_0.2_	1.0	48.0±_0.1_	63.8±_0.1_
15	43.6±_0.2_	59.4±_0.1_	**24.4%**	**50.3**±_0.1_	**65.9**±_0.2_	[0.3, 0.3, 0.2, 0.2]	45.3±_0.2_	61.0±_0.2_	140	49.1±_0.0_	64.5±_0.0_	**1.2**	**50.3**±_0.1_	**65.9**±_0.2_
20	42.6±_0.1_	58.3±_0.1_	42.2%	41.0±_0.1_	56.8±_0.0_	[0.4, 0.4, 0.1, 0.1]	42.2±_0.2_	57.8±_0.2_	160	47.0±_0.0_	62.5±_0.1_	1.4	49.5±_0.2_	65.2±_0.1_

**Table 10:** Ablation analysis of different graph projection choices and masking strategies used in our proposed Vox-ZSDA on Poly-GA dataset. “Graph CL” is short for the graph contrastive learning strategy. $\ell_{2}$ and $\ell_{\infty }$ denote the Euclidean distance and Chebyshev distance, respectively

Source macromolecules: 1bxn, 1f1b, and 1yg6					
Distance Metrics	mIoU	Dice	Graph CL	mIoU	Dice
$\ell_{2}$	41.8	58.2	Random	37.8	53.0
**Ours** $\left(\ell_{\infty }\right)$	**46.9**	**62.4**	**Ours (Dynamic)**	**46.9**	**62.4**

## Footnotes

1 Datasets are released via https://figshare.com/s/72204297b23906ce5163
